# Lung influenza virus-specific memory CD4 T cell location and optimal cytokine production are dependent on interactions with lung antigen-presenting cells

**DOI:** 10.1016/j.mucimm.2024.06.001

**Published:** 2024-10

**Authors:** Kerrie E. Hargrave, Julie C. Worrell, Chiara Pirillo, Euan Brennan, Andreu Masdefiol Garriga, Joshua I. Gray, Thomas Purnell, Edward W. Roberts, Megan K.L. MacLeod

**Affiliations:** 1 Centre for Immunobiology, School of Infection and Immunity, University of Glasgow, UK; 2 Cancer Research UK Scotland Institute, Glasgow, UK; 3 Centre for Virus Research, School of Infection and Immunity, University of Glasgow, UK

## Abstract

Influenza A virus (IAV) infection leads to the formation of mucosal memory CD4 T cells that can protect the host. An in-depth understanding of the signals that shape memory cell development is required for more effective vaccine design. We have examined the formation of memory CD4 T cells in the lung following IAV infection of mice, characterizing changes to the lung landscape and immune cell composition. IAV-specific CD4 T cells were found throughout the lung at both primary and memory time points. These cells were found near lung airways and in close contact with a range of immune cells including macrophages, dendritic cells, and B cells. Interactions between lung IAV-specific CD4 T cells and major histocompatibility complex (MHC)II+ cells during the primary immune response were important in shaping the subsequent memory pool. Treatment with an anti-MHCII blocking antibody increased the proportion of memory CD4 T cells found in lung airways but reduced interferon-γ expression by IAV-specific immunodominant memory CD4 T cells. The immunodominant CD4 T cells expressed higher levels of programmed death ligand 1 (PD1) than other IAV-specific CD4 T cells and PD1+ memory CD4 T cells were located further away from MHCII+ cells than their PD1-low counterparts. This distinction in location was lost in mice treated with anti-MHCII antibodies. These data suggest that sustained antigen presentation in the lung impacts the formation of memory CD4 T cells by regulating their cytokine production and location.

## Highlights

• Lung influenza A virus (IAV)-specific cluster of differentiation (CD)4 T cells are located near major histocompatibility complex (MHC)II+ cells during the primary response and following viral clearance.
• Interactions between CD4 T cells and MHCII+ cells in the lung during the primary response to IAV decrease the proportion of memory CD4 T cells found in lung airways.
• Lung IAV-specific memory CD4 T cells expressing high levels of programmed death ligand 1 are less likely to be in close proximity to MHCII+ cells than programmed death ligand 1 low memory CD4 T cells.

## Graphical abstract

**Figure:**
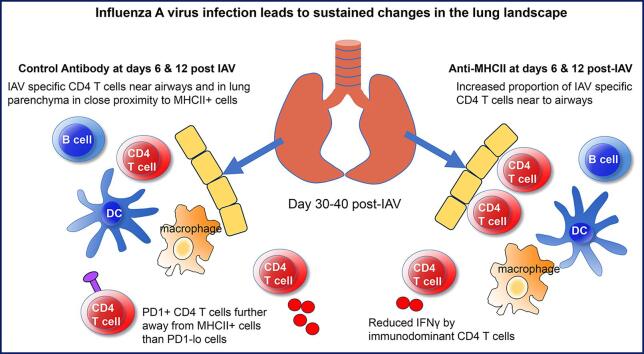

## INTRODUCTION

Influenza A virus (IAV) is a common pathogen that infects the respiratory tract. Despite the availability of vaccines, IAV continues to present a substantial global health challenge. Current vaccines generate an antibody response that targets the highly variable surface proteins of the virus. While antibodies can neutralize the virus, seasonal viral mutation and immune selection make it challenging for antibodies to recognize modified viruses.[1], [2] The leads to viral immune evasion, the outbreak of seasonal epidemics, and the risk of pandemics.

In contrast to antibodies, cluster of differentiation (CD)4 T cells often recognize viral regions conserved between different IAV strains.^3^ Following IAV infection, activated CD4 T cells migrate to the lung where they play diverse roles in viral control including helping CD8 T cells and B cells, in addition to mediating direct effector functions via cytokines.[4], [5] Some of these recruited CD4 T cells will remain in the lung after infection as tissue-resident memory (Trm) cells. Lung memory CD4 T cells can provide enhanced protection from re-infection compared to memory cells from secondary lymphoid tissues by acting directly and through interactions with other cells to limit viral replication.[6], [7], [8] Importantly, the presence of IAV-specific CD4 T cells correlates with reduced disease symptoms in humans.[9], [10]

Typically, mouse Trm cells are examined by flow cytometry based on their persistence within the tissue in parabiosis experiments, expression of certain retention molecules, including CD69 or CD103 and/or Trm-related transcription factors, and lack of binding to antibodies injected into the blood shortly before analysis.[11], [12] Parabiosis experiments can provide definitive information on tissue residency, although are complex experiments that are neither possible nor desirable for routine analysis. Other methods provide a snapshot of where the T cell is and what it is expressing at the time of analysis.

While there is a growing understanding of the heterogeneity of Trm cells^13^^,^^14^ much less is known about the location of Trm within the lung and whether this alters following resolution of the infection. Here we have used reporter mice to identify lung IAV-specific CD4 T cells at multiple time points following infection. We identified IAV-specific CD4 T cells dispersed throughout the lung, in airways, and within clusters with other immune cells, including various types of major histocompatibility complex (MHC)II+ antigen-presenting cells (APCs). While the composition of the APCs altered during the infection time course, CD4 T cells were found in close proximity to MHCII+ cells at both primary and memory time points.

The close proximity between IAV-specific CD4 T cells and these APCs indicates a role for antigen presentation in the generation of memory T cells. Inhibiting the interactions between CD4 T cells and APCs with a blocking anti-MHCII antibody can disrupt early T cell responses and/or the formation of immune memory depending on the timing of delivery.[15], [16], [17] Here we have delivered anti-MHCII intranasally (i.n.) following the recruitment of CD4 T cells to the lung to investigate the role of interactions between lung CD4 T cells and APCs in the generation of immune memory.

We found that the numbers of IAV-specific memory CD4 T cells were not altered by anti-MHCII treatment. However, the location of IAV-specific memory CD4 T cells and their ability to produce the key anti-viral cytokine, interferon (IFN)γ, were altered by the blockade. We found that anti-MHCII treatment increased the percentage of memory cells found in airways but reduced IFNγ production by CD4 T cells that recognize an immunodominant IAV nucleoprotein (NP) epitope. Together, these data indicate that interactions between lung APCs and CD4 T cells influence the quality and location of memory CD4 T cells generated by infection, findings that have important implications for the design of mucosal vaccines.

## RESULTS

### IAV infection leads to sustained changes in the lung

**Fig. 1:**
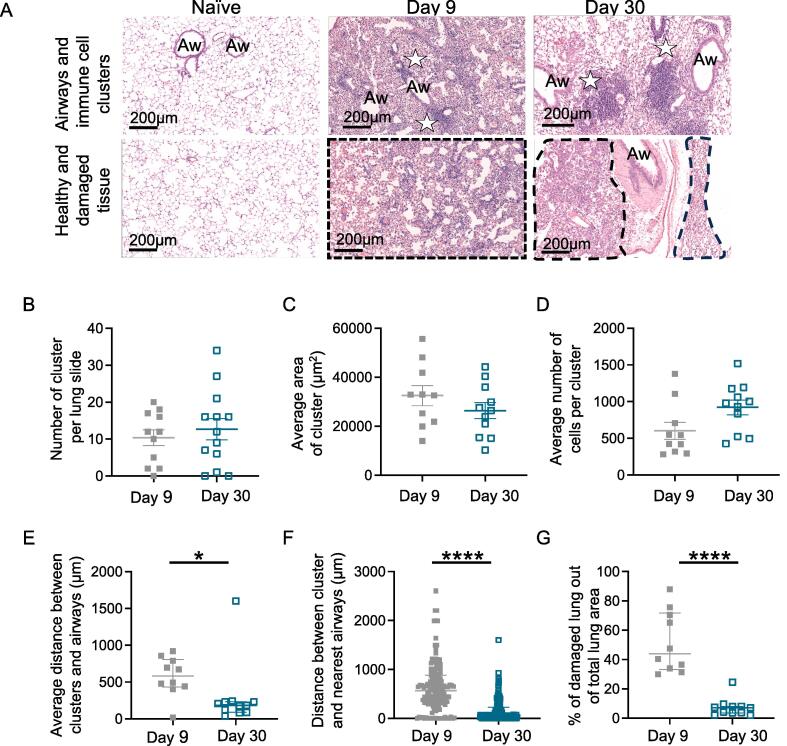
Persistent changes to the lung landscape following IAV infection. C57BL/6 mice were infected intranasally with IAV and lungs taken from naive animals or at 9 or 30 days post-infection. (A) H&E staining on lung sections from naive and IAV-infected mice; all images taken at 10x magnification, scale bar 200 µm. Airways, immune cell clusters (white stars), and areas of damage (black dashed areas) are indicated. Blinded analysis of lung sections was performed using ImageScope software to determine (B) the number of areas of high cell density clusters; (C) average measurement of the cluster area (µm^2^); (D) average number of cells contained within a cluster; (E) average and (F) individual distance/proximity between clusters and the nearest airway (µm); (G) percentages of damaged lung areas; in (C–F), 1 day 9 and 2 day 30 samples were removed as no clusters were present. In (B–E, and G) each symbol represents a mouse and the line is the mean of the group (B–D) or median (E, G). In (F), each symbol represents a cluster, and the line shows the median of all clusters. Error bars are SEM (B–D) or show the interquartile range (E–G). Data are from two experiments with five or six mice/experiment taken at day 9, and two experiments with six or seven mice/experiment taken at day 30, each slide contained one section of each of the five lung lobes, and data shown are added from across all lobes to provide one value per slide. In (E, F, and G) data are not normally distributed (tested by Shapiro-Wilk) and were analyzed by Mann-Whitney *U* test, *p < 0.05; ***p < 0.001; ****p < 0.0001. Aw = airways; H&E = hematoxylin and eosin; IAV = influenza A virus; SEM = standard error of the mean.

To first characterize how influenza virus infection alters the landscape of the lung, we performed a blinded analysis of hematoxylin and eosin (H&E) stained lung sections taken at days 9 and 30 following infection with IAV (strain WSN) (Fig. 1A). H&E-stained sections containing a section from each lung lobe were digitally scanned, and a blinded analysis was performed to identify IAV-induced alterations in the lung. Lungs from infected mice had clear areas of cell infiltrate including dense cell clusters, often found near airways and that are similar to structures described by others.[18], [19], [20], [21], [22], [23], [24]

We determined the number of clusters per lung, their size, cell number, and distance from the nearest airway. We identified an average of 10.4 clusters per lung section at day 9 and 12.7 at day 30 post-infection (Fig. 1B). These data demonstrate that clusters are maintained despite viral clearance and formation of immune memory.[25], [26], [27] The cluster area and number of cells within the clusters also did not alter significantly between the primary and memory time points (Fig. 1C and D). However, the clusters tended to be closer to airways at memory time points when we calculated on a per animal or per cluster basis (Fig. 1E and F). At both primary and memory time points, tissue sections contained areas of damage, often located near the immune cell clusters. To calculate the percentage of the lung that contained such tissue, areas of damage that were not the cell clusters were expressed as a percentage of the total lung area (Fig. 1G). This measure reduced from the primary to the memory time point, consistent with repair to the lung following viral clearance.^28^

### Immune cells are found throughout the lung, near airways, and in cell clusters at primary and memory time points following IAV infection

**Fig. 2:**
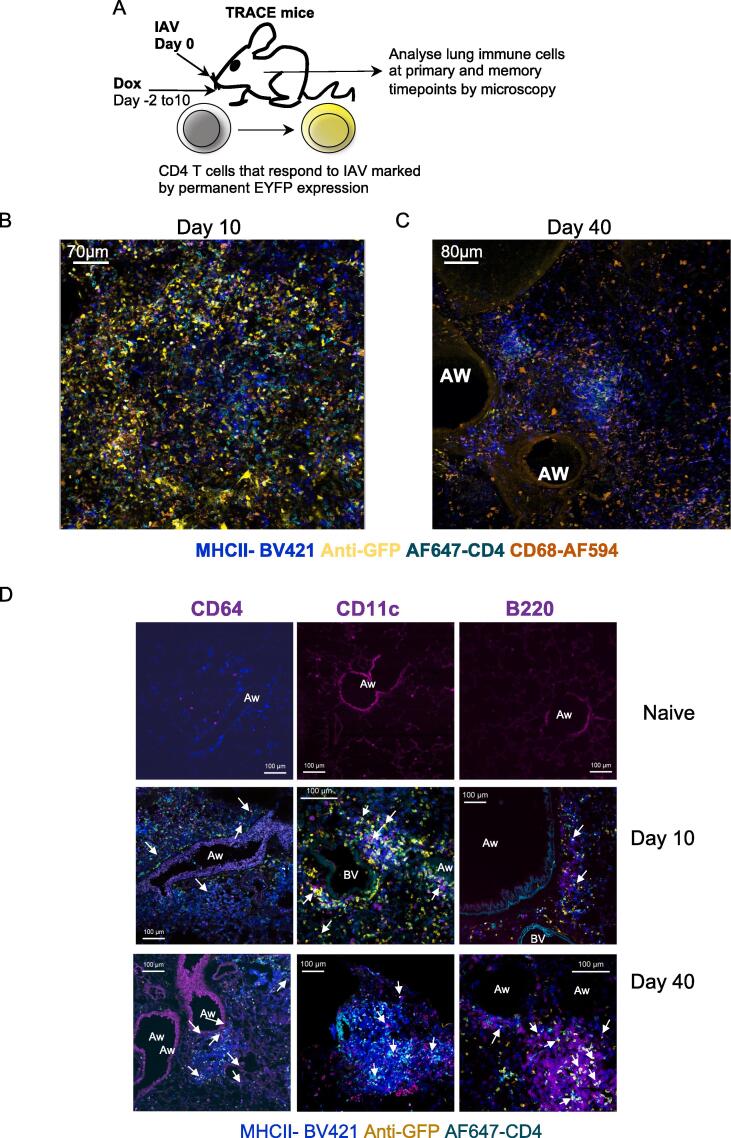
Immune cells are found throughout the lung, near airways, and in cell clusters at primary and memory time points following IAV infection. (A) TRACE mice enable identification of responding CD4 T cells via permanent expression of EYFP. Lungs from naive or IAV-infected TRACE mice were analyzed 10 or 40 days post-IAV infection. In (B–C), PCLS were cleared and stained with the indicated antibodies, scale bars are 70 μm (A) or 80 μm (B). In (D), frozen lungs were sectioned and stained with the indicated antibodies to identify IAV-specific CD4 T cells, and MHCII+ CD64, CD11c, or B220+ cells. Data are representative of one image set (B–C) or three (naive), four (B220, CD64), or 6–7 (CD11c) mice per time point from two experiments (D). White arrows indicate EYFP+CD4+ cells in close proximity to CD64, CD11c, or B220+ cells, scale bars are 100 μm. CD = cluster of differentiation; EYFP = enhanced yellow fluorescent protein; IAV = influenza A virus; MHC = major histocompatibility complex; PCLS = precision-cut lung slices; TRACE = T cell Reporter of Activation and Cell Enumeration.

To investigate the location of immune cells within the lung parenchyma, airways, and clusters, we performed immunofluorescence analysis of lungs at days 10 and 40 post-infection. To first provide an overview of the lung immune response, we combined precision-cut lung slices (PCLS) with tissue clearing and confocal microscopy. To enable us to identify IAV-specific CD4 T cells, we took advantage of a reporter mouse, T cell Reporter of Activation and Cell Enumeration (TRACE), that we have developed and previously characterized.[29], [30] Activated T cells in these triple transgenic mice express rtTA under the control of part of the interleukin 2 promoter. When bound to doxycycline, rtTA activates the tet-ON promoter driving Cre expression. This leads to the irreversible labeling of activated T cells by expression of the enhanced yellow fluorescent protein (EYFP) from the Rosa locus (Fig. 2A). Infection with IAV induces a robust population of EYFP+ CD4 T cells when reporter mice are fed doxycycline in their diet during the initial infection.^30^ Our prior studies have shown that EYFP+ cells are found in lung and secondary lymphoid organs following IAV infection.^30^ This model greatly increases the number of responding T cells we can identify by both flow cytometry and immunofluorescence in comparison to other methodologies such as T cell receptor (TCR) transgenic cells or MHC tetramers. Moreover, it enables us to identify a broad range of responding CD4 T cells rather than identifying cells that respond to a single epitope.

The cleared lung tissue showed that multiple immune cell populations were distributed throughout the parenchyma at day 10 post-infection (Fig. 2B). In contrast, at day 40 post-infection, immune cells were more obviously packed together in clusters, often around airways and similar to the structures we observed in the H&E analysis (Fig. 2C).

We next used confocal microscopy of lung sections to more closely examine the cells within the clusters and the cells located near airways. A large number of cells were MHCII+ and we stained sections with antibodies to CD64, B220, and CD11c to provide insights into the identity of these cells (Fig. 2D and Supplementary Fig. 1). MHCII+ cells that also expressed either CD64, B220, and CD11c were identified in close proximity to IAV-specific CD4 T cells at both time points. The representative images demonstrate the diversity of the anti-viral response with some images illustrating tight networks of immune cells clustered together while others show immune cells forming a line around airways.

As multiple cell types can express CD64, CD11c, and B220, we used flow cytometry to identify and characterize different MHCII+ subsets in naive animals and at primary and memory IAV infection time points. We first used the gating strategy in Supplementary Fig. 2A to define multiple populations of myeloid or lymphoid cells and assessed the changes in the proportion of these cells across the time points. We then gated on MHCII+ cells that were either CD64+, B220+, or CD11c+ (Supplementary Fig. 2B) and overlaid the initial gates to identify which cell types were within these MHCII+ populations.

**Fig. 3:**
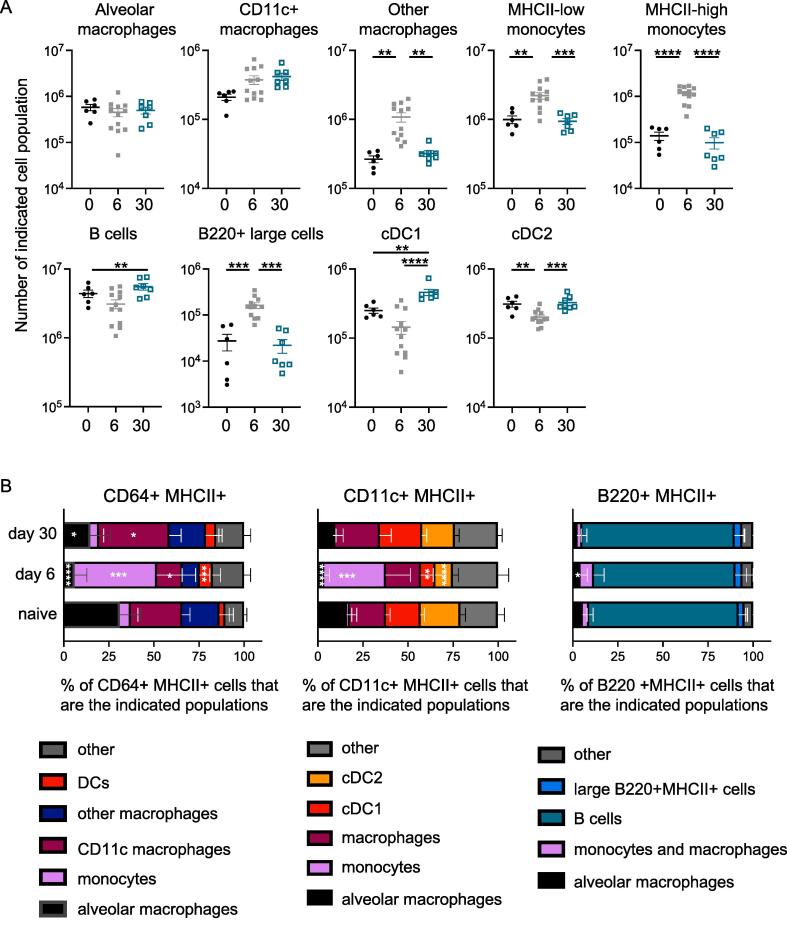
Lung MHCII+ populations alter across the IAV infection time course. C57BL/6 mice were infected with IAV, and lungs taken at 6 or 30 days post-infection or from naive animals. Following tissue digest, lungs were analyzed by flow cytometry as indicated in Supplementary Fig. 2. In (A), each symbol represents a mouse and the line shows the mean of the group. Data are from two independent experiments per time point with a total of 6–12 per group: naive: three mice/experiment; day 6: six mice/experiment; day 30: three and four mice/experiment. All data were normally distributed (tested by Shapiro-Wilk) and differences between time points assessed by ANOVA followed by a Tukey’s test. In (B), cells were gated on MHCII+ cells that were CD64, CD11c, or B220+, and the proportion of the indicated cell populations determined. Not all data were normally distributed and differences between infection time points and naive samples tested by ANOVA followed by a Dunn’s multiple comparison test. In (A), the horizontal line is the mean, and error bars are SEM. In (B), error bars are SD, and in (A–B) *p < 0.05; **p < 0.01; ***p < 0.001; ****p < 0.0001. ANOVA = analysis of variance; CD = cluster of differentiation; IAV = influenza A virus; MHC = major histocompatibility complex; SD = standard deviation; SEM = standard error of the mean.

As expected,[31], [32] during the initial response, there was an increase in monocyte and macrophage populations in the lung (Fig. 3A). There were fewer lung CD103+ conventional (c) DCs at day 6 post-infection compared to in naive animals, indicating the migration of these cells to the draining lymph node.[33], [34] Most of these populations returned to levels found in naive animals at the memory time point. However, we did find a sustained increase in the numbers of B cells and CD103+ cDC1 at day 30 post-IAV infection.

In line with these changes at the cell population level, CD11c+MHCII+ cells and CD64+MHCII+ cells contained a greater proportion of monocytes and macrophages at day 6 post-infection (Fig. 3B). In these analyses, we have combined related populations, e.g. different macrophage populations, when one or both populations were present at low frequencies. By day 30, the proportional make-up of these populations was similar to that in naive animals although the CD64+MHCII+ population contained slightly fewer alveolar macrophages and slightly more CD11c+ macrophages at day 30 compared to naive animals. In contrast, the populations of B220+MHCII+ cells were consistent across the time course and were mainly B cells. Together, these data suggest that multiple different types of MHCII+ cells have the potential to interact with CD4 T cells in the lung during and following IAV infection.

### IAV-specific CD4 T cells are found in the airway at primary and memory time points

**Fig. 4:**
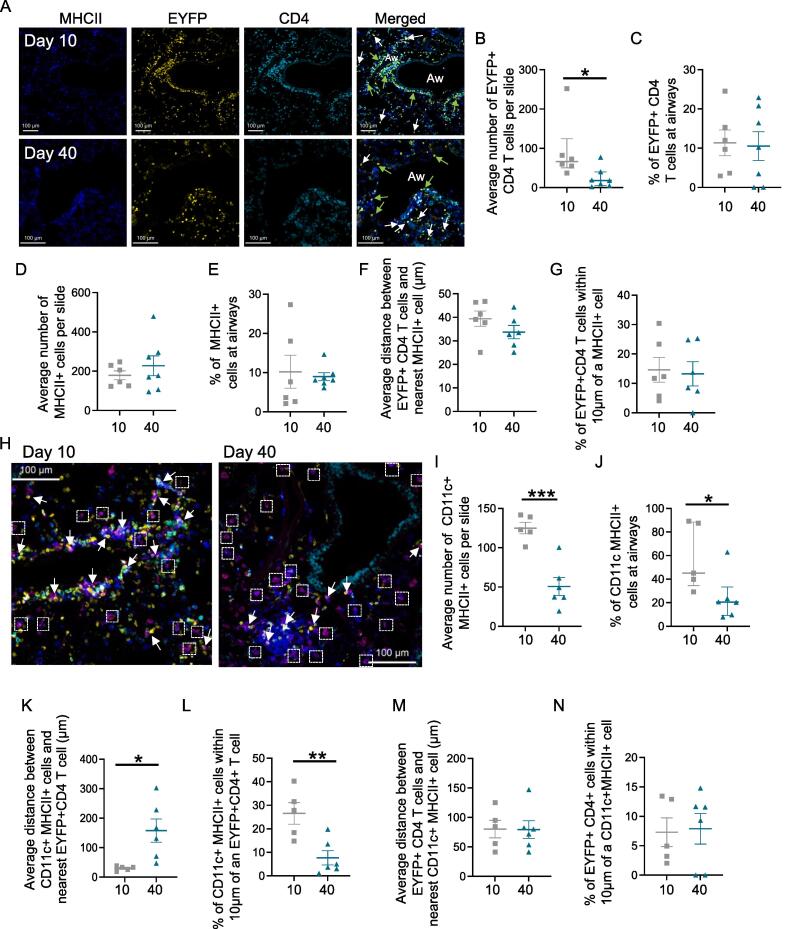
IAV-specific CD4 T cells are maintained in lung airways at memory time points. TRACE mice were infected with IAV, and lungs taken 10 or 40 days post-infection. Lung sections were stained with the indicated antibodies to identify MHCII+ cells and IAV-specific CD4 T cells. In (A and H), images are representative of 6–7 mice (day 10: three mice from two experiments; day 40: three or four mice/experiment). In (A), green and white arrows show EYFP+CD4+ cells near or further away from airways, respectively. In (H), arrows indicate CD11c+MHCII+ cells next to EYFP+CD4+ cells and boxes indicate cells more than  20 μm away from CD4+EYFP+ cells. In A and H scale bars are  10 μm. In (B–G) and (I–N), each symbol represents a mouse from the same experiments and the line shows the mean of the group, error bars are SEM (C–G and I, K–N) or median and interquartile range, (B, J). In (B and J), data are not normally distributed (tested by Shapiro-Wilk), and difference tested between time points tested by a Mann-Whitney *U* test, data in (I, K, and L) were normally distributed and differences tested by t tests. In all: *p < 0.05; **p < 0.01; ***p < 0.001. CD = cluster of differentiation; EYFP = enhanced yellow fluorescent protein; IAV = influenza A virus; MHC = major histocompatibility complex; SEM = standard error of the mean; TRACE = T cell Reporter of Activation and Cell Enumeration

CD4 T cells are known to persist in the lung following IAV infection^4^^,^^5^ and we have characterized the number, phenotype, and cytokine production by IAV-specific CD4 T cells in previous studies.[29], [30], [35], [36] Similar to the MHCII+ cells, IAV-specific CD4 T cells were found in the lung parenchyma, at airways, and in immune clusters at primary and memory time points (Fig. 2, Fig. 4A). As expected, the numbers of EYFP+CD4+ cells found per slide declined from day 10 to day 40 post-infection (Fig. 4B). Despite this, the percentages of these cells that were found at the airways were consistent between the two time points suggesting that memory CD4 T cells are just as likely to patrol the sites of infection during and beyond an active infection (Fig. 4C). In contrast, the numbers of MHCII+ cells and the percentage of these cells found at airways did not change between the primary and memory time points (Fig. 4D and E).

We measured the distance between the EYFP+CD4 T cells and the nearest MHCII+ cell to ask whether the likelihood of cell interactions altered between the primary and memory time points. The average distance to the nearest MHCII+ cells was the same at both time points (Fig. 4F). We also asked what percentage of the EYFP+CD4+ cells were within 10 μm of the nearest MHCII+ cell. The distances measured are from cell centroid to centroid, and thus cells within 10 μm or less of each other are likely to be touching and potentially interacting. At primary and memory time points, a mean of 11% and 13% respectively of the EYFP+CD4+ cells were within 10 mm of an MHCII+ cell (Fig. 4G). Together, these data suggest that IAV-specific T cells maintain similar levels of contact with APCs at the primary and memory time points.

As our flow cytometry data showed that the numbers of CD103+ cDC1s remained elevated at 30 days post-infection, we examined the location of CD11c+MHCII+ cells at primary and memory time points. The number of CD11c+ MHCII+ cells found per slide did decline between day 10 and day 40 (Fig. 4H and I and Supplementary Fig. 3). This difference with the flow data likely reflects the more precise cell identification possible by flow compared to microscopy. Unlike the EYFP+CD4 T cells, fewer CD11c+MHCII+ cells were found at airways at day 40 compared to day 10 post-infection (Fig. 4J). Additionally, the distance between CD11c+MHCII+ cells and the nearest EYFP+ CD4 T cells increased from day 10 to day 40, and, consistently, the percentage of CD11c+MHCII+ cells within 10 μm of a EYFP+CD4 T cells declined (Fig. 4H, K, and L). Consistent with the microscopy data on total MHCII+ cells, measurements from the perspective of EYFP+CD4 T cells showed a similar distance between these cells and CD11c+MHCII+ cells at the two time points (Fig. 4M). Similarly, the percentage of EYFP+CD4+ cells within 10 μm of a CD11c+MHCII+ was also equivalent between day 10 and day 40 (Fig. 4N). Together, these data show that IAV-specific memory CD4 T cell location and proximity to APCs are consistent between the primary and memory timepoints. In contrast, there is limited evidence that, as a population, CD11c+MHCII+ location is permanently altered by the infection.

### Immunodominant but not polyclonal IAV-specific memory CD4 T cells have a reduced IFNγ response following TCR-MHC blockade

Our imaging studies indicated that lung IAV-specific CD4 T cells are likely to have multiple interactions with APCs during IAV infection. In mice, IAV is cleared within the first 10 days of an infection[26], [27] but antigen can be presented to CD4 T cells for at least 35 days post-infection.^37^ We wanted to investigate whether presentation of antigen in the lung was important in the generation of memory CD4 T cell function and location. To address this, we delivered an MHCII blocking antibody^16^ i.n. at days 6 and 12 post-infection. These time points were chosen to allow an initial T cell response to be primed and to block TCR peptide MHC (pMHC) interactions during the peak response in the lung.[25], [36] Importantly, the antibody treatment did not alter the infection-induced weight loss (Supplementary Fig. 4).

First, to confirm that the anti-MHCII bound to the different APC populations in the lung, we used fluorescently labeled antibodies and examined the lung MHCII+ populations after 2 hours or 2 days. The antibody rapidly bound all MHCII+ cells in the lung including monocytes, macrophages, B cells, and DCs (Supplementary Figs. 5A–C). In contrast, an isotype control antibody bound to only a small proportion of cells. Some i.n. instilled anti-MHCII labeled cells could be found in the draining lymph node 2 hours after instillation, however, only a small percentage (5.7 ± 7.4) of the MHCII+ cells were labeled (Supplementary Fig. 5D).

Two days after the instillation, the anti-MHCII was still present in the different APC populations, in particular all lung B cells, CD103+ cDC1, and DC2 cells were positive for the antibody (Supplementary Fig. 6A and B). Antibody treatment had a limited effect on the recruitment of MHCII+ cells, although we did find an increased number of cDC2 in some anti-MHCII treated animals (Supplementary Fig. 6C).

While more cells were positive for the instilled anti-MHCII in the draining lymph at day 2 than at 2 hours following instillation, the intensity of staining with the antibody was much lower than that in the lung (Supplementary Fig. 6D, F–H). Very few spleen cells were positive for the instilled anti-MHCII 2 days after the instillation (Supplementary Fig. 6E, I–K). Together, these data show that the instilled anti-MHCII antibody binds strongly to lung MHCII+ cells but does not saturate MHCII molecules on draining lymph nodes or spleen MHCII+ cells.

**Fig. 5:**
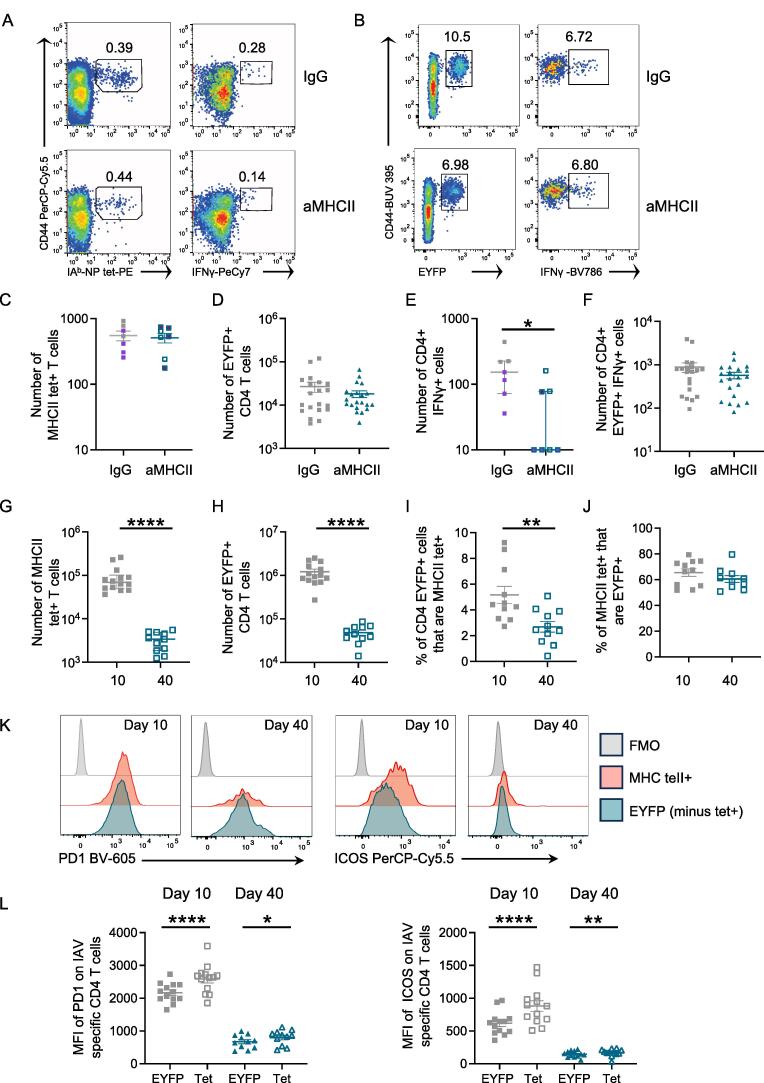
Immunodominant but not polyclonal IAV-specific memory CD4 T cells have a reduced IFNγ response following anti-MHCII treatment. C57BL/6 (A, C, E) or TRACE (B, D, F) mice were infected with IAV on day 0 and given control IgG or anti-MHCII intranasally on days 6 and 12. On day 40, IAV-specific CD4 T cells were detected *ex vivo* using IA^b^/NP_311-325_ tetramers (C) or following restimulation with NP_311-325_ peptide (E) or co-culture with IAV-Antigen treated bmDCs (D, F). In (G–L), TRACE mice were infected with IAV and CD4+EYFP+ cells and MHCII tetramer+ examined following 10 or 40 days. In (A–B), cells are gated on CD4+ cells as shown in Supplementary Fig. 7 and the numbers are the percent of cells in the indicated gate, in (K–L) cells are gated shown in Supplementary Fig. 8. In (A, C, E) data are from two experiments with 3/4 mice/experiment and colored symbols indicate the two experiments; in (B, D and F) data are from five experiments with the following numbers of mice/group: IgG, 3, 2, 5, 5, 5; anti-MHCII: 4, 4, 4, 4, 5. In (G–L), mice are from two experiments with: day 10: four or seven mice/group; day 40: four or eight mice/group. Symbols show the mean of the group and error bars are SEM. In (E), data are not normally distributed (tested by Shapiro-Wilk), and difference tested by Mann-Whitney *U* test, the Y axis is set at the level of detection. In (G), data are not normally distributed and tested via a Mann-Whitney *U* test. In (H–L), data are normally distributed, and differences tested using a t test in (H–J) and paired t tests between cell types in (K–L). In all graphs, *p < 0.05; **p < 0.01; ***p < 0.001; ****p < 0.0001. bmDC = bone marrow-derived dendritic cell; CD = cluster of differentiation; EYFP = enhanced yellow fluorescent protein; IAV = influenza A virus; IFN = interferon; Ig = immunoglobulin; MHC = major histocompatibility complex; NP = nucleoprotein; SEM = standard error of the mean; TRACE = T cell Reporter of Activation and Cell Enumeration.

To investigate the consequences of the anti-MHCII treatment, we used flow cytometry to identify IAV-specific CD4 T cells that recognize the immunodominant NP peptide, 311-325, or by gating on EYFP+ CD4 T cells in infected TRACE mice (Fig. 5A and B), gating in Supplementary Fig. 7. Our prior study demonstrated similar kinetics and differentiation into cytokine-producing cells between CD4 T cells identified via these two methods.^30^

There were no differences in the numbers of either IA^b^/NP_311-325_ tetramer (tet)+ or EYFP+ CD4 T cells between animals treated with anti-MHCII or immunoglobulin (Ig)G control antibody (Fig. 5C and D). To examine production of the key T cell-derived anti-viral cytokine, IFNγ, we restimulated the lung cells *ex vivo* with NP_311-325_ or bone marrow-derived dendritic cells (bmDCs) incubated with sonicated IAV antigens.[30], [35] While anti-MHCII treatment reduced the number of NP_311-325_ specific IFNγ+ CD4 T cells within the memory pool, there was no difference in the number of EYFP+ IFNγ+ in infected TRACE mice (Fig. 5E and F). These data suggest there may be different requirements for sustained antigen presentation in the formation of memory CD4 T cells specific for different IAV epitopes.

### Lung immunodominant IAV-specific CD4 T cells express higher levels of T cell activation markers than polyclonal IAV-specific T cells

To explore the difference in dependency on TCR-pMHCII interactions on the generation of IFNγ+ memory cells, we examined the phenotype of these cells in IAV-infected TRACE mice. As expected, the numbers of both CD4 T cells detected by MHCII tetramer and EYFP+ CD4 T cells declined from day 10 to day 40 post-IAV infection (Fig. 5G and H). When we examined the percentages of EYFP+ cells that were also MHCII-tet+, we found this declined from day 10 to day 30 post-infection (Fig. 5I). These data indicate that CD4 T cells specific for NP_311-325_ may be less likely to enter the memory pool than other IAV-specific CD4 T cells. In contrast, the percentages of MHCII-tet+ cells that were also EYFP+ were consistent at both time points (Fig. 5J).

We examined expression of programmed death ligand 1 (PD1) and Inducible T cell costimulator (ICOS), two molecules increased following sustained TCR activation, and that are expressed by some memory T cells, including some IAV-specific lung memory CD4 T cells.[13], [14], [30], [38] Additionally, in a previous study,^30^ we found that cells with a highly differentiated phenotype expressed high levels of both molecules, suggesting that PD1 and ICOS expression marks T cells that have received strong and/or prolonged TCR activation signals.

As expected, memory CD4 T cells expressed lower levels of both molecules than cells at day 10 post-infection. However, at both time points, MHCII tet+ CD4 T cells expressed higher levels of PD1 and ICOS in comparison to the remainder of the EYFP+ population (Fig. 5K and L and Supplementary Fig. 8). We found a similar pattern of expression in CD4 T cells from the lung draining lymph node and spleen at day 10 post-infection but the difference at the memory time point was minimal or absent (Supplementary Fig. 9). Together these data suggest that, particularly in the lung, CD4 T cells specific for an immunodominant IAV epitope express higher levels of molecules associated with T cell activation than other IAV-specific CD4 T cells. This distinction may explain their reduced entry into the memory pool and greater dependence on antigen presentation compared to other IAV-specific CD4 T cells.

### Anti-MHCII treatment alters the proportion of memory IAV-specific CD4 T cells in the airways

**Fig. 6:**
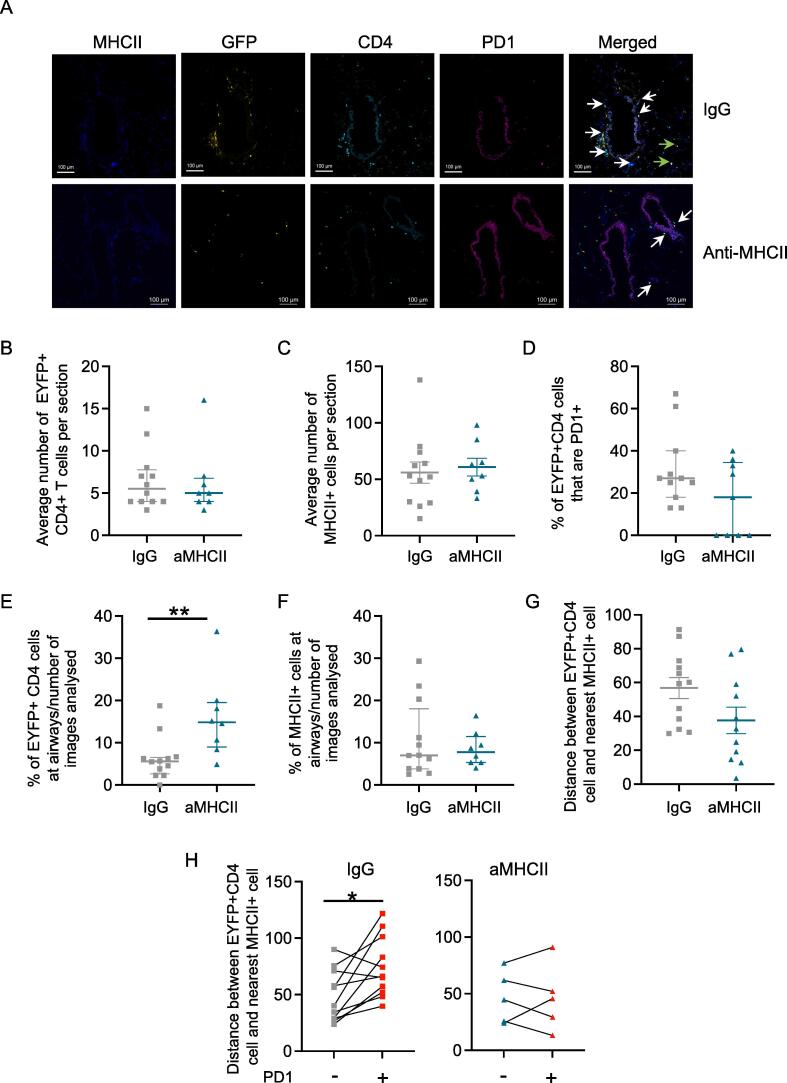
Anti-MHCII treatment alters the location of IAV-specific CD4 T cells. TRACE mice were infected with IAV on day 0 and on days 6 and 12 given control IgG or anti-MHCII intranasally. On day 40, lungs were frozen and tissue sections stained with the indicated antibodies. Data are combined from two independent experiments: IgG: 12 mice:6/experiment; anti-MHCII: eight mice 4/experiment. In (A), white and green arrows indicate EYFP+CD4+ cells and EYFP+CD4+PD1+ cells respectively; scale bar is 100 μm. In (B–G), each symbol represents a mouse, the line shows the mean of the group, and error bars are SEM. For each mouse 2–5 sections were analyzed. In (B, D, E and F) data are not normally distributed (tested by Shapiro-Wilk) and differences tested by Mann-Whitney *U* test. In (C, G and H), data are normally distributed and differences tested by t test (C, G) or Paired t test (H). In (H), four anti-MHCII mice are removed as no EYFP+CD4+ PD1+ cells were found in the analyzed sections. CD = cluster of differentiation; EYFP = enhanced yellow fluorescent protein; IAV = influenza A virus; Ig = immunoglobulin; MHC = major histocompatibility complex; PD1 = programmed death ligand 1; SEM = standard error of the mean; TRACE = T cell Reporter of Activation and Cell Enumeration

To investigate whether the location of IAV-specific memory CD4 T cells was altered following anti-MHCII treatment, we examined lung sections by immunofluorescence at day 40 post-infection. As in the flow cytometry experiments, mice were treated with anti-MHCII or control IgG i.n. at days 6 and 12 post-infection. It is not possible to use MHCII tetramers on frozen sections. However, we examined the location of MHCII+ cells and EYFP+ CD4 T cells and determined the expression of PD1 by the CD4 T cells (Fig. 6A).

In line with our flow cytometry data, neither the numbers of EYFP+CD4 T cells nor MHCII+ cells identified per slide were altered by the antibody treatment (Fig. 6B and C). There was a slight but non-significant reduction in the percent of EYFP+ CD4 T cells that were PD1 positive and in some anti-MHCII treated mice, we could not detect any PD1+ EYFP+ CD4 T cells (Fig. 6D).

To investigate whether the anti-MHCII treatment affected CD4 T cell location, we assessed whether the EYFP+ CD4 T cells were proximal to airways. We normalized for the number of slides with airways by dividing the percentages of EYFP+CD4 cells at airways by the number of slides that had airways. These data show that blocking MHCII-TCR interactions during the primary immune response led to an increase of memory CD4 T cells in airways (Fig. 6E). There was not, however, any impact on the location of MHCII+ cells themselves (Fig. 6F).

We also measured the distances between the EYFP+ CD4 T cells and the nearest MHCII+ cell. Anti-MHCII treatment led to a slight but non-significant decline in the distance between the EYFP+ CD4 T cells and nearest MHCII+ cell (Fig. 6G). More strikingly, there was a clear difference in the distance between EYFP+CD4 T cells and the nearest MHCII+ cell depending on the T cell’s expression of PD1. EYFP+ CD4 T cells that were PD1+ were further away from MHCII+ cells than PD1 negative T cells (Fig. 6H). These data suggest that the PD1 expression might not be a consequence of a recent interaction with an APC. In contrast, in anti-MHCII treated animals in which we could detect PD1+ cells, this distinction in distance to the nearest MHCII+ cell was lost.

To explore this difference in cell location further, we examined the expression of molecules associated with Trm cells^11,^^12^ and the inflammatory chemokine receptor, CXCR3, by flow cytometry in IAV-infected TRACE mice treated with control IgG or anti-MHCII (Supplementary Fig. 10). Few EYFP+CD4 T cells expressed CD103 but expression of CD69 was highest on lung EYFP+CD4 T cells. In all organs, the majority of EYFP+CD4 T cells expressed CXCR3, with over three-quarters of the lung EYFP+CD4+ T cells positive for this chemokine receptor. The expression of these molecules was not affected by the anti-MHCII treatment suggesting that factors other, or in addition to these, influence the location of memory CD4 T cells in the lung.

Taken together with the results in Section 3.4, these data suggest that interactions between CD4 T cells in the lung during the later stages of the primary response are required to shape important attributes of memory CD4 T cells. Antigen-driven interactions may be required for optimal cytokine responses by some T cells. In contrast, these same interactions may reduce the opportunity for the CD4 T cells to position themselves near airways, often the initial infection site for respiratory viruses.

## DISCUSSION

Heterogeneity within the memory CD4 T cell pool has focused on cell phenotype, gene expression, and location.[39], [40] Memory cell location has mainly been examined at the organ level, comparing cells within lymphoid and non-lymphoid tissues. Here we focused on the micro-location of lung IAV-specific memory CD4 T cells determining how the location of these cells alters from the primary to the memory phase of the response. Our data demonstrate that both primary and memory CD4 T cells are found in multiple micro-locations including at airways and in packed clusters with other immune cells. Importantly, our data show that local TCR-pMHC signals delivered during the later stages of the primary immune response are not required for the formation of memory cells in the lung. However, these signals do influence the ability of immunodominant CD4 T cells to produce IFNγ and limit the access of memory CD4 T cells to airways.

We analyzed IAV-driven changes to the lung using H&E and confocal analysis of immune cells. In both cases, our data demonstrate persistent changes to the landscape of the lung following infection. This is consistent with long-term changes to the lung following IAV infection in mice and sustained symptoms in some clinical infections.[41], [42], [43], [44]

Clusters of immune cells that persist for months following IAV infection have been documented previously.[19], [20], [21] The clusters in our studies do not have clear B and T cell zones and thus we have not referred to them as induced bronchus-associated lymphoid tissue or tertiary lymphoid organs.[22], [23] We found a wide range of immune cells within clusters including those expressing B220, CD64, and CD11c. The B220+ cells are likely B cells, known to be retained in the lung and important for protection from re-infection, in particular following challenge with the same IAV strain.^20^ The IAV-specific CD4 T cells contained within clusters with the B220+ cells are likely to overlap with tissue resident helper cells that can promote protection from a different IAV strain.[13], [14]

IAV-specific CD4 T cells were found in close proximity to MHCII+ cells at both primary and memory time points following infection. These data suggest that the CD4 T cells may continue to interact with APC despite viral clearance. IAV antigen can be maintained in the lung and/or lung draining lymph nodes for weeks/months following infection [37], [45] and these interactions may shape the survival and subsequent function of memory CD4 T cells.

We found that IAV-specific CD4 T cells were maintained at airways from the primary to the memory response. Moreover, we increased the proportion of IAV-specific CD4 T cells in airways by treating the mice with a blocking anti-MHCII antibody during the late primary response. These data indicate that interactions between CD4 T cells and APCs at this time shape the subsequent memory pool. Lung airway epithelial cells are some of the first cells to be infected with IAV^46^ and by positioning themselves near airways, memory CD4 T cells have the potential to respond rapidly following a re-infection, either in an antigen-specific manner via presentation by an APC, including epithelial cells,[47], [48] or potentially, in response to local cytokines.^49^

It is unclear why interactions with lung APCs would limit airway memory CD4 T cells. One potential explanation is that interactions between the CD4 T cells and MHCII+ epithelial^47^ cells may limit the T cells ability to persist in the airways. Alternatively, sustained interactions could drive increased effector cell differentiation, and that these highly differentiated cells present a risk of immune-driven damage at delicate airways.[50], [51] This hypothesis would fit with our finding that anti-MHCII treatment reduced IFNg production by immunodominant, but not all, IAV-specific memory CD4 T cells. It is likely that the immunodominant T cells have proliferated substantially during the infection, having received strong activation signals, corresponding with our finding that they express higher levels of PD1 and ICOS than the majority of IAV-specific CD4 T cells. Interestingly, lung IAV-specific memory CD4 T cells expressing PD1 were more likely to be further away from MHCII+ cells than those with low PD1 expression. These data suggest that cells that receive strong activation signals may be restricted from future interactions by limiting their access to MHCII APCs. An alternative explanation is that PD1 expression indicates cells that have recently received a TCR signal and that these cells have migrated away from the APC following a productive interaction.

Together, these data suggest that interactions between lung CD4 T cells and APCs temper, but are not required for, the generation of memory cells. These data highlight the value of analyzing responding T cells using multiple readouts to provide a more complete understanding of the molecular regulation of memory cell formation. As the anti-MHCII antibody bound to many types of cells and the IAV-specific CD4 T cells could be found near to multiple MHCII+ cells, it was not possible to identify which APCs may be involved in tempering the generation of memory CD4 T cells. Potentially, all the different MHCII+ cells could influence memory CD4 T cell generation.

In summary, this study extends our understanding of heterogeneity within the lung memory CD4 T cell pool. CD4 T cells within and outside lung immune clusters will experience different signals and interact with different cell types suggesting distinct memory niches that may influence the type and function of lung memory CD4 T cells.^52^ It will be key for future studies to understand the dynamics of cells within these different environments. For example, to address whether cells can move into and out of clusters, what controls these decisions during the maintenance of immune memory during, and, importantly, how these cells respond to successive infections.

## METHODS

### Animals

Ten-week-old female C57BL/6 mice were purchased from Envigo (United Kingdom) and male and female TRACE mice were bred at the University of Glasgow. TRACE and C57BL/6 mice were maintained at the University of Glasgow under specific pathogen-free conditions in accordance with UK home office regulations (Project Licenses P2F28B003 and PP1902420) and approved by the local ethics committee. TRACE mice have been described previously.[29], [30] No formal randomization was performed and sample size was based on previous studies. In some experiments, samples were removed as cell numbers were too small or lymph nodes not found as indicated in relevant figure legends.

### IAV infections

TRACE or C57BL/6 mice were briefly anesthetized using inhaled isoflurane and infected with 100–200 plaque-forming units of IAV WSN strain in 20 µL of phosphate-buffered saline (PBS) i.n. depending on their age, weight, and sex. IAV was prepared and titered in Madin-Darby Canine Kidney cells (MDCK). Infected mice were weighed daily from day 4 post-infection. Any animals that lost more than 20% of their starting weight were humanely euthanized. TRACE mice were given Dox+ chow (Envigo) for a total of 12 days starting 2 days prior to infection. Cages with mice that lost weight, were given soft diet until all mice returned to their starting weight or above.

### Intranasal antibody instillation

Mice were anesthetized using inhaled isoflurane at days 6 and 12 post-infection and 100 μg of anti-MHCII (Y3P) or rat IgG2a (2A3), both from BioXcell (USA), instilled in 30μl of PBS. For Y3P labeled studies, Y3P and IgG were labeled with Alexa-Fluor 546 (ThermoFisher) according to the manufacturer’s instructions. Mice received the labeled antibody on day 6 post-infection and were euthanized after either 2 hours or 2 days. Control IgG and anti-MHCII treatments were given to mice within the same cages to prevent cage-specific effects acting as confounders.

### Digital slide scanning

Excised lung tissues for histological analysis were fixed in 10% neutral buffered formalin for 24 hours and paraffin-embedded in a tissue processor (UK, Shandon Pathcentre Tissue Processor). Formalin-fixed and paraffin-embedded tissues were sectioned (6 μm) and processed for subsequent staining with H&E using standard protocols. Stained slides were scanned on a Leica Aperio VERSA 8 bright-field slide scanner (Leica Biosystems, UK) at 10X magnification and were visualized/analyzed using Aperio ImageScope version 12.1.0.5029 (Aperio Technologies Inc., Vista, USA).

### Blinded analysis of H&E staining

Manual identification of selected regions of interest (ROI) was performed by a blinded observer. ImageScope software (Aperio Technologies Inc) was used to provide independent outputs for each tissue section. This included quantifying the number of areas of high cell density (hematoxylin stained, clusters), measurement of the cluster area (expressed as µm^2^) measurement of distance/proximity between clusters and the nearest airway (expressed as µm), and assessment of damaged area (expressed as % of total lung area). The Aperio nuclear V9 algorithm (Aperio ScanScope XT System; Aperio Technologies) was used to count the number of cells contained within a cluster. Damaged areas were identified as ROIs that were not airways, blood vessels, immune cell clusters, or normal/healthy lung tissue as found in the naive animals.

### Immunofluorescent imaging

Mice were euthanized by rising concentrations of carbon dioxide (CO_2_) and the vena cava cut. Lungs were perfused with PBS-5 mM ethylenediaminetetraacetic acid to remove red blood cells and 1% paraformaldehyde was used to fix the lungs and preserve the integrity of the EYFP protein. Lung inflation was achieved using 1–3 mL 1% warm UtraPure low melt agarose administrated via the trachea. Lungs with solidified agarose were incubated in 1% paraformaldehyde overnight at 4 °C followed by incubation in 30% sucrose for a further 2–5 days at 4 °C. Lung lobes were frozen in Optimal Cutting Temperature (OCT) (Tissue-Tek 4583, UK) and stored at −80 °C. Lungs were sectioned into 10 μm slices on a Shandon Cryotome FE (Thermo Scientific 12087159, UK) and mounted onto Super Frost slides (Thermo Scientific). Slides were fixed in 100% cold acetone and stored at −20 °C.

Slides were rehydrated in PBS containing 0.5% bovine serum albumin (BSA) for 5 minutes and incubated with Fc block (24G2) for 30–60 minutes at room temperature. The sections were stained with antibodies at 4 °C overnight: anti-CD4 AlexaFluor647 (BD UK, RM4-5), anti-MHCII eFluor 450 (M5114, ThermoFisher, UK), anti-B220-Phycoerythrin (PE) (ThermoFisher, RA3-6B2), anti-CD64-PE (BioLegend, UK, X54-5/7.1), anti-CD11c-Alexa-594 (BioLegend, N418), anti-PD1-PE (BioLegend, 29F.1A12), anti-green fluorescent protein (GFP)-Alexa 488 (rabbit polyclonal, Invitrogen, UK). Slides were washed in PBS-BSA and mounted with VectorSheild (Vector, UK). Images were collected on an LSM880 (Zeiss, Germany) confocal microscope at 20× magnification.

For PCLS, lungs inflated with agarose were sectioned at 300 μm using a vibrotome (5100 mz Campden Instruments, UK) and stored at 4 °C in PBS containing 1% BSA and 0.05% sodium azide. Sections were incubated with protein block (Abcam) before the addition of primary antibodies: anti-CD4 AlexaFluor647 (BD, RM4-5), anti-MHCII eFluor 450 (M5114, ThermoFisher), anti-CD68 AF594 (FA-11, BioLegend) and anti-GFP-Alexa 488 (rabbit polyclonal, Invitrogen, UK) diluted in antibody diluent reagent solution (Life Technologies, UK). The slices were incubated overnight at 4 °C and then washed with wash buffer (1% BSA, 0.1% TritonX-100, and 0.05% sodium azide in PBS). Ce3D clearing solution (Biolegend) was added 30 minutes before mounting in a seal frame incubation chamber (ThermoFisher) and covered with a coverslip. Images were acquired using a Zeiss LSM 880 Airyscan confocal microscope and analyzed using Imaris software Oxford Instruments, UK.

### Image analysis

Confocal slide images were prepared in Zen (Zeiss) and analyzed in Volocity (Version 6, Quorum Technologies, Canada). Objects were defined based on fluorescence, object size, and pixels, in cases of high cell density, touching objects were separated. Cells were manually checked when used for measurement analysis. Distances were measured using the measuring tool and ROIs around airways cropped to count cells at airways.

### Tissue preparation for CD4 T cell analysis by flow cytometry

Prior to euthanasia by cervical dislocation, TRACE mice were injected intravenously with 1 μg PE-conjugated anti-CD45 (clone; 30F11, ThermoFisher) for 3 minutes. Alternatively, in the C57BL/6 mouse experiments in Fig. 5, mice were euthanized using a rising concentration of CO_2_ and perfused with PBS with 5 mM ethylenediaminetetraacetic acid through the heart. In both cases, single-cell suspensions of lungs were prepared by digestion of snipped lung tissue with 1 mg/mL collagenase and 30 μg/mL DNase (Sigma) for 40 minutes at 37 °C in a shaking incubator and tissues disrupted by passing through a 100 mm filter. Spleen and mediastinal lymph nodes were processed by mechanical disruption. Red blood cells were lysed from spleen and lungs with lysis buffer (ThermoFisher). Cells were counted using a hemocytometer with dead cells excluded using Trypan Blue.

### Tissue preparation for APC analysis by flow cytometry

Mice were euthanized by cervical dislocation and lungs digested with a final concentration of 1.6 mg/mL Dispase, 0.2 mg/mL collagenase P (Roche, UK) and 0.1 mg/mL DNase (Sigma, UK) for 40 minutes at 37 °C in a shaking incubator and tissues disrupted by passing through a 100 μm filter. Red blood cells were lysed. Cells were counted using a hemocytometer with dead cells excluded using Trypan Blue.

### Production of bone marrow-derived dendritic cells

Bone marrow-derived dendritic cells were generated from femurs and tibias of 8–10-week-old C57BL/6 mice with Roswell Park Memorial Institute medium (RPMI) 1640 (supplemented with 10% heat-inactivated fetal calf serum, 100 mg/μL penicillin-streptomycin and 2 mM L-glutamine). Cells were cultured at 37 °C 5% CO_2_ in the presence of Granulocyte-Macrophage Colony Stimulating Factor (GM-CSF) (conditioned from the supernatant of X63 cells^53^) for 7 days. Media was supplemented or replaced on days 2 and 5. After this time, adherent cells were harvested, seeded, and then incubated overnight with IAV antigen (multiplicity of infection of 0.3) produced as described previously.^35^

### *Ex vivo* reactivation for cytokine production

Lung single-cell suspensions were co-cultured with IAV-Antigen+ bmDCs in complete RPMI at a ratio of approximately 10:1 T cells to DCs. Alternatively, cells were cultured with 10 μg/mL NP peptide (QVYSLIRPNENPAHK, JPT, Germany). *Ex vivo* cultures were incubated at 37 °C, 5% CO_2_ for 6 hours in the presence of Golgi Plug (BD Bioscience).

### Flow cytometry staining

#### IAV-specific CD4 T cells

When required, single-cell suspensions were stained with PE or APC-labeled IA^b^/NP_311-325_ tetramers (National Institute of Health (NIH) tetramer core, USA) for 2 hours at 37 °C, 5% CO_2_ in complete RPMI containing Fc block (24G2). After this time, surface antibodies were added and the cells incubated for a further 20 minutes at 4 °C. Alternatively, cells were incubated for 10 minutes at 4 °C with Fc block and surface antibodies added for a further 20 minutes. Antibodies used were: anti-CD4 APC-Alexa647 (RM4-5, ThermoFisher), anti-CD8 BUV805 (BD 53-6.7) or anti-CD8-eFluor 450 (ThermoFisher 53-6.7), anti-CD44 PerCP-Cy5.5 or BUV395 (ThermoFisher or BD: IM7), anti-PD1 BV605 (BioLegend 29F.1A12), anti-ICOS PerCP-Cy5.5 (ThermoFisher 7E.17G9), anti-B220 eFluor 450 (RA3-6B2), anti- MHCII eFluor 450 (M5114) and anti- F480 eFluor 450 (BM8), all ThermoFisher. Anti-B220, MHCII and F480, and CD8, were used as a ‘dump’ gate. Cells were stained with a fixable viability dye eFluor 506 (ThermoFisher).

For some experiments, cells were fixed with cytofix/cytoperm (BD Bioscience) for 20 minutes at 4 °C and stained in perm wash buffer with anti-IFNγ PE or BV785 (ThermoFisher or BioLegend, clone: XMG1.2) for 1 hour at room temperature.

#### Lung and lymphoid APCs

Cells were first labeled with Fc block for 10 minutes at 4 °C and then the following antibodies added: CD45-PE (BD, 30-F.11), Siglec F-APC (BioLegend, S17007L), CD11b-PeCy7 (BioLegend, M1/70), Ly6G-BV785 (BioLegend, 1A8), Ly6C-PerCP-Cy5.5 (ThermoFisher, HK1.4), MHCII BUV395 (BD, 2G9), CD64-BV711 (BioLegend, X54-5/7.1), CD11c-eFluor780 (ThermoFisher, N418), B220-eFluor 450 (RA3-6B2, ThermoFisher), CD103-Qdot 605 (BioLegend, 2E7), CD3-FITC (145-2C11, BioLegend), CD8α APC (53-6.6, ThermoFisher). Cells were then stained with a fixable dye eFluor 506 (ThermoFisher).

Stained cells washed with FACS buffer or Permwash following intracellular staining and acquired on a BD Fortessa and analyzed using FlowJo (version 10 BD Bioscience, USA).

### Statistical analysis

Data were analyzed using Prism version 10 (GraphPad, USA). Differences between groups were analyzed as indicated in figure legends depending on whether data were normally distributed, tested via Shapiro-Wilk test and the numbers of groups analyzed. In all figures * represents a *p* value of <0.05; **p > 0.01, ***p > 0.001, ****p > 0.0001.

## DECLARATION OF COMPETING INTEREST

The authors have no competing interest.

## FUNDING

The work was supported by a Marie Curie Fellowship (334430), a Wellcome Trust Investigator Award (210703/Z/18/Z) to MKLM, and a Rosetrees’ Trust Seedcorn Grant (Seedcorn2020\100017) awarded to JCW.

## ACKNOWLEDGMENTS

We thank the staff within the School of Infection and Immunity Flow Cytometry Facility, the Glasgow Imaging Facility, and Biological Services at the University of Glasgow for technical assistance. We thank Pablo Murcia for helpful conversations on viral responses. We thank the National Institutes of Health tetramer core facility for the provision of IA^b^-NP_311-325_ tetramers.

## DATA AVAILABILITY

All raw data are available upon request to the corresponding author.

## CRediT authorship contribution statement

**Kerrie E. Hargrave:** Writing – review & editing, Writing – original draft, Visualization, Investigation, Formal analysis, Data curation, Conceptualization. **Julie C. Worrell:** Writing – review & editing, Writing – original draft, Visualization, Investigation, Funding acquisition, Formal analysis, Data curation. **Chiara Pirillo:** Writing – review & editing, Investigation, Formal analysis. **Euan Brennan:** Writing – review & editing, Investigation, Formal analysis. **Andreu Masdefiol Garriga:** Writing – review & editing, Investigation, Formal analysis. **Joshua I. Gray:** Writing – review & editing, Investigation, Formal analysis. **Thomas Purnell:** Writing – review & editing, Investigation. **Edward W. Roberts:** Writing – review & editing, Formal analysis. **Megan K.L. MacLeod:** Writing – review & editing, Writing – original draft, Visualization, Project administration, Investigation, Funding acquisition, Formal analysis, Conceptualization.
