# Supplementary material for: Lung influenza virus-specific memory CD4 T cell location and optimal cytokine production are dependent on interactions with lung antigen-presenting cells
Source: Mucosal Immunol. 2024 Oct;17(5):843–57. doi: 10.1016/j.mucimm.2024.06.001 (PMC11464401; doi:10.1016/j.mucimm.2024.06.001)
Supplement: Supplementary Data 1 [file mmc1.pdf]

Supplementary Figure 1: Identification of MHCII+ cells in IAV infected lungs at primary and memory timepoints

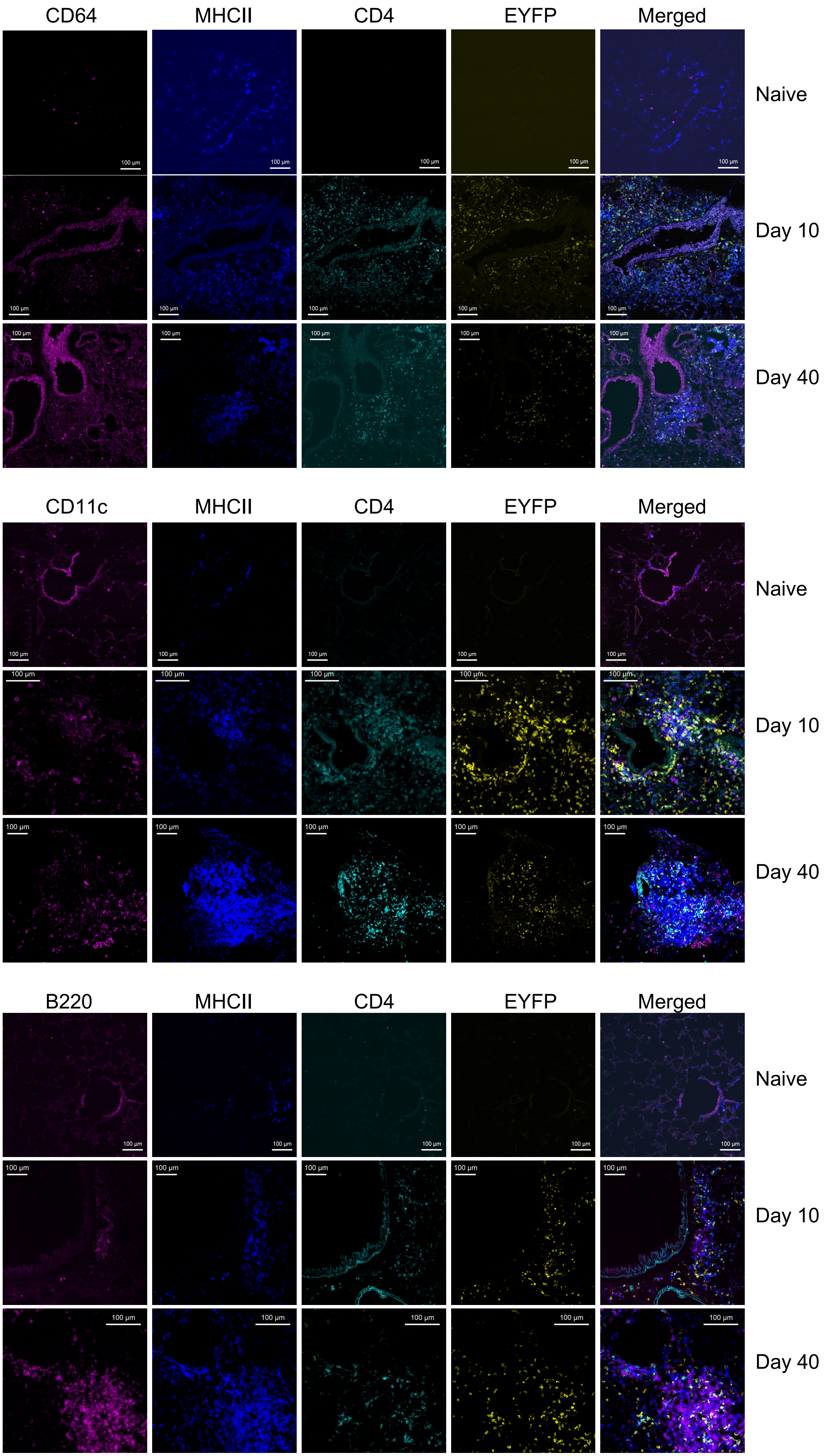

Supplementary Figure 1. Identification of MHCII+ cells in IAV infected lungs at primary and memory timepoints

TRACE mice were either naïve or infected with IAV and lungs removed 10 or 40 days post-infection. Lungs were sectioned and stained with the indicated antibodies to identify IAV specific CD4 T cells and MHCII+ CD64, CD11c or B220+ cells. Data are representative 3 (naïve), 4 (B220, CD64) or 6-7 (CD11c) mice per time point from two experiments and scale bars are 100μm.

Supplementary Figure 2: Gating strategy to identify MHCII+ populations

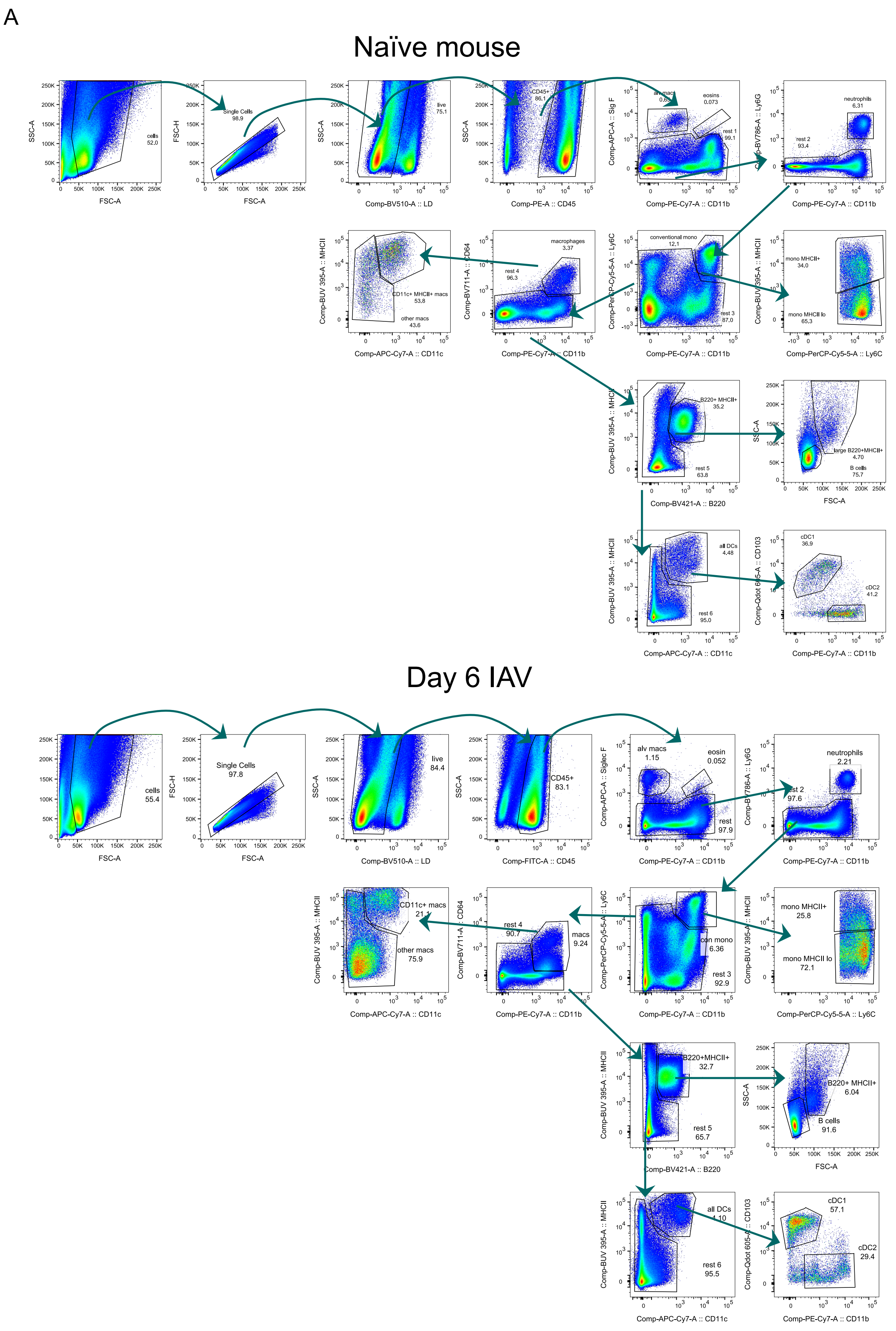

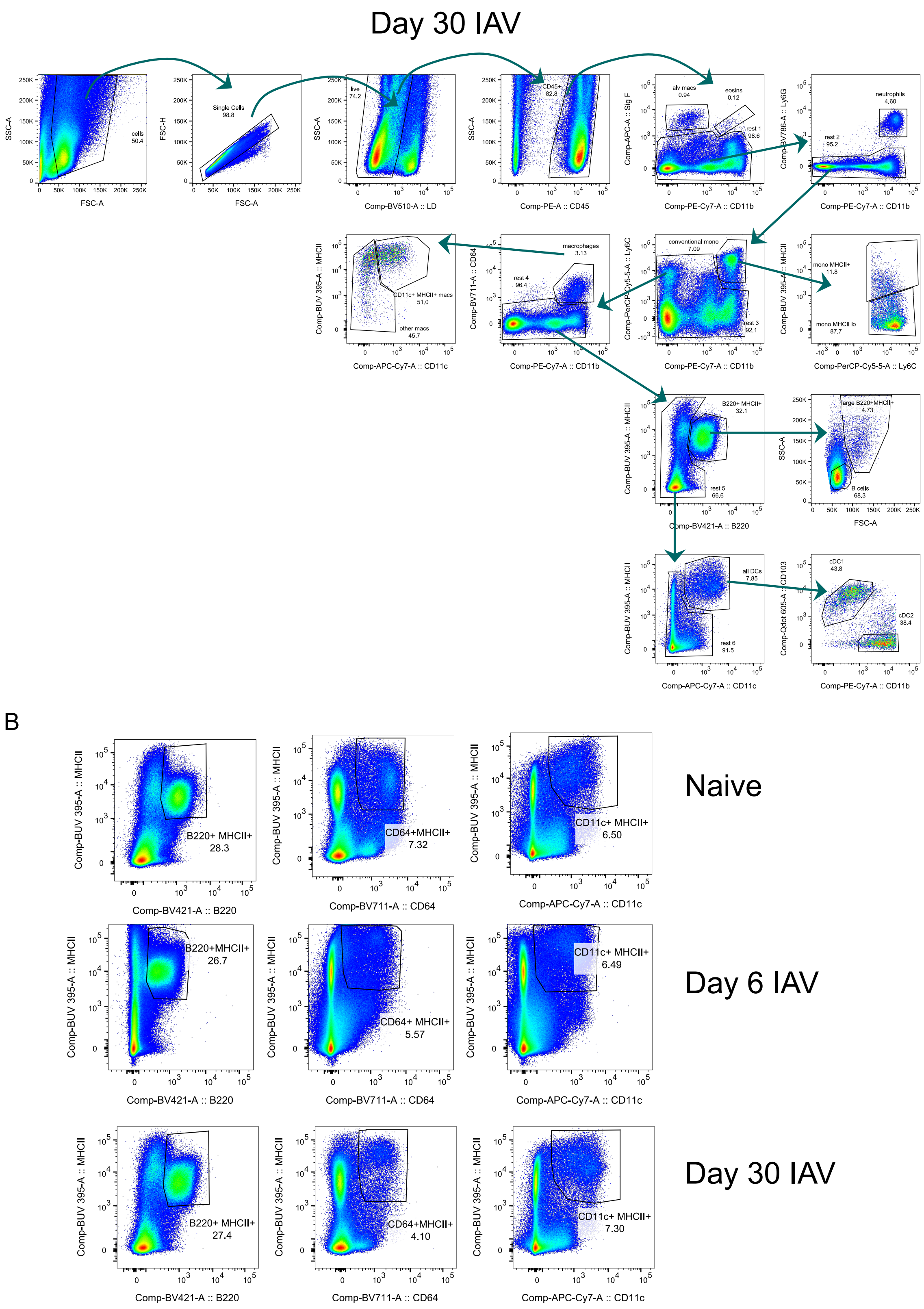

Supplementary Figure 2. Gating strategy to identify MHCII+ populations

Flow cytometry analysis of lungs from naïve mice or mice infected with IAV 6 or 30 days earlier. A shows example gating. In B, cells from the same samples are first gated through the CD45+ gate in A to identify populations of MHCII+ B220, CD64 and CD11c+ cells. Naïve and day 30 mice from the same experiment, day 6 mouse from a separate experiment. Data are representative of 2 independent experiments per time point with a total of 6-12 per group: naive: 3 mice/experiment; day 6: 6 mice/experiment; day 30: 3 and 4 mice/experiment

Supplementary Figure 3: Individual images for data in Figure 4

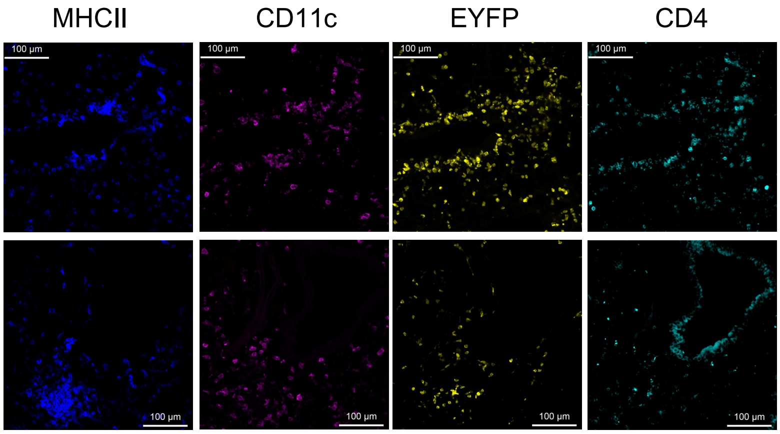

Supplementary Figure 3. Individual images for CD11c data shown in Figure 4

TRACE mice were infected with IAV and lungs taken 10 or 40 days post-infection. Lung sections were stained with the indicted antibodies to identify MHCII+ cells and IAV specific CD4 T cells, images are representative of 5-6 mice from 2 experiments.

Supplementary Figure 4: Anti-MHCII treatment does not alter IAV-induced weight loss

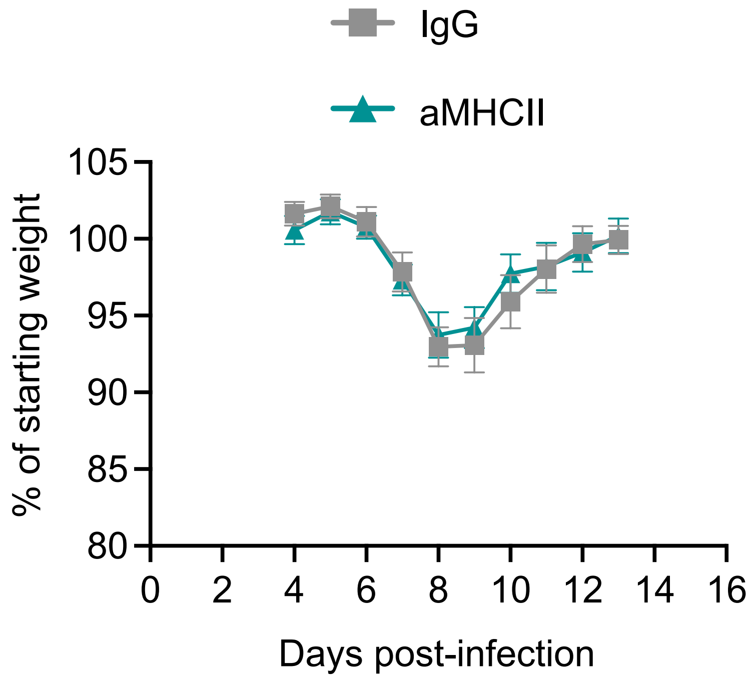

Supplementary Figure 4. Anti-MHCII treatment does not alter IAV-induced weight loss

TRACE mice were infected with IAV on day 0 and weighed from day 4 until day 14 post-infection. Mice received 100µg of either control IgG or anti-MHCII intranasally on day 6 and 12 post-infection. Data are combined from 5 experiments with a total of 22 (IgG) and 22 (anti-MHCII) animals per group with 3-5 mice from each experiment.

Supplementary Figure 5. Intranasal anti-MHCII binds to MHCII+ cells in the lung but to few MHCII+ cells in the draining lymph node 2 hours post-instillation

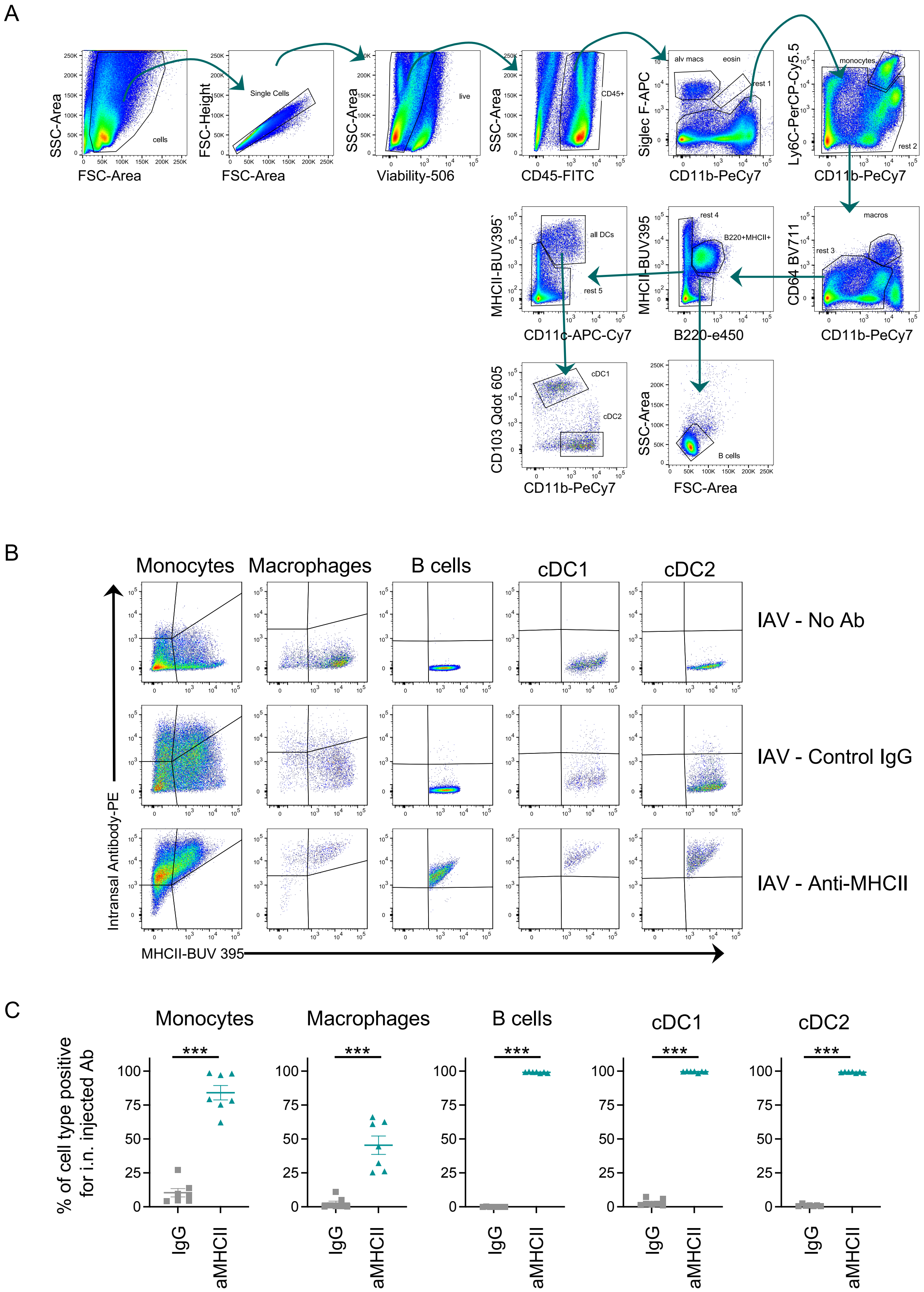

D

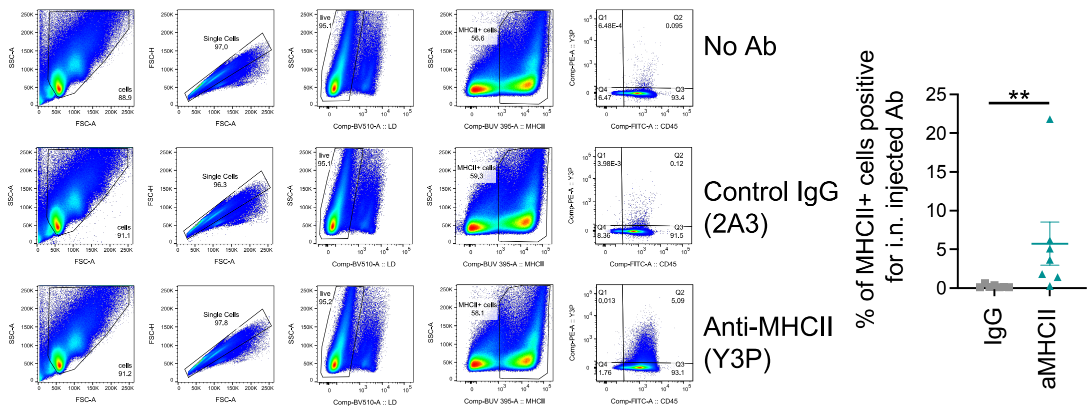

Supplementary Figure 5. Intranasal anti-MHCII binds to MHCII+ cells in the lung but to few MHCII+ cells in the draining lymph node 2 hours post-instillation

C57BL/6 mice were infected with IAV i.n. on day 0 and received 100µg of either control IgG or anti-MHCII labelled with Alexa-Fluor546 i.n on day 6. 2 hours later, single cell suspensions of lung (A-C) and mediastinal lymph node cells (D) were stained for flow cytometry. The gating strategy in A was used to identify lung monocytes, macrophages, B cells, cDC1 and cDC2. The data are representative of two experiments with a total of 8 (control IgG, 4/experiment) and 7 (anti-MHCII, 3-4/experiment) mice. In C and D, the percentages of the cell populations double positive for the i.n. antibody and anti-MHCII labelled *ex vivo* are shown, error bars are SEM and the horizontal line shows the mean of the group. Data were not normally distributed (tested by Shapiro-Wilk), significance tested with a Mann Whitney U test, **:p<0.01 ***: p<0.001.

Supplementary Figure 6. Intranasal anti-MHCII binds to MHCII+ cells in the lung more robustly than to MHCII+ cells in the dLN or spleen 2 days post-instillation

A

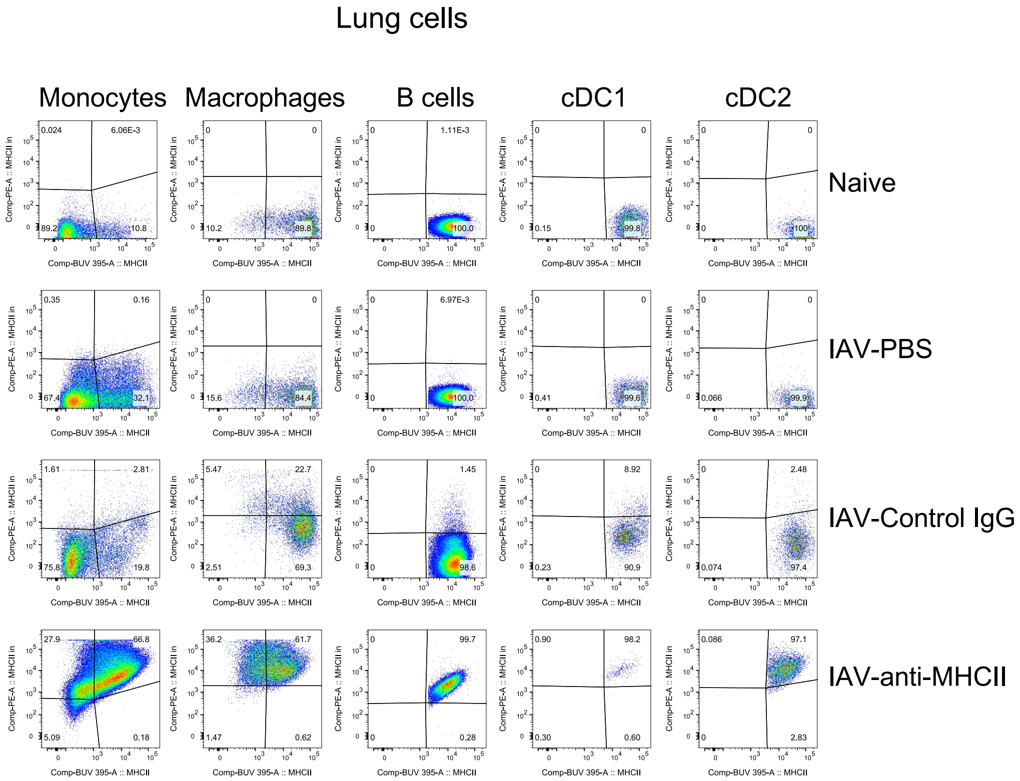

B

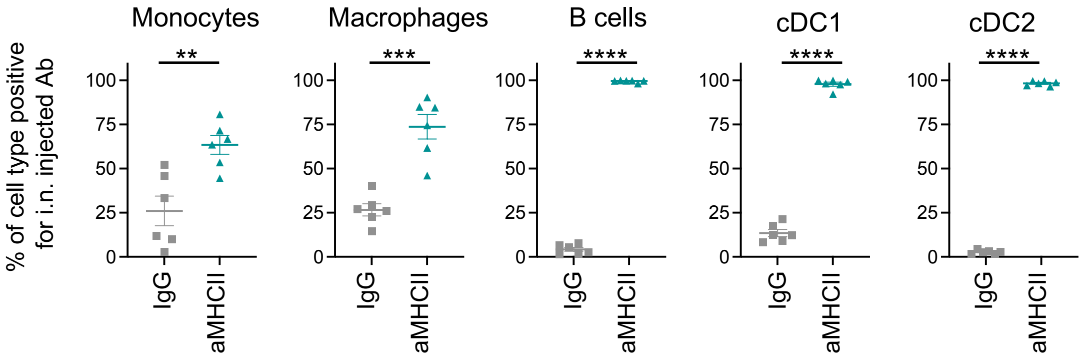

C

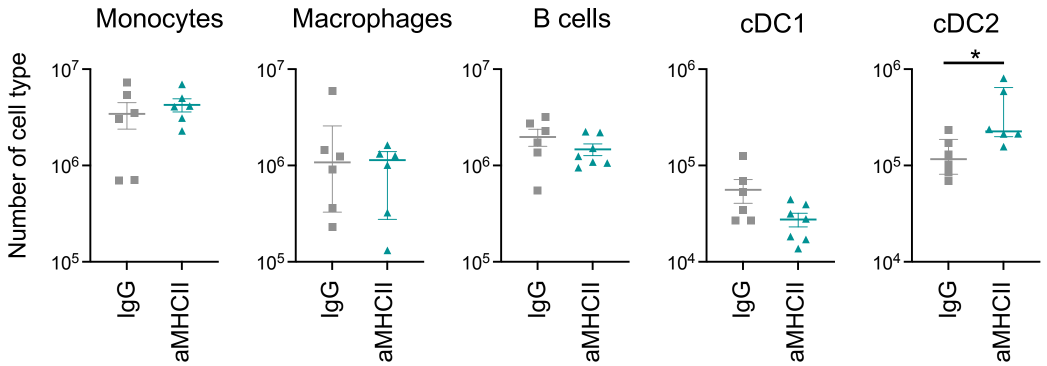

D

Draining lymph node gating

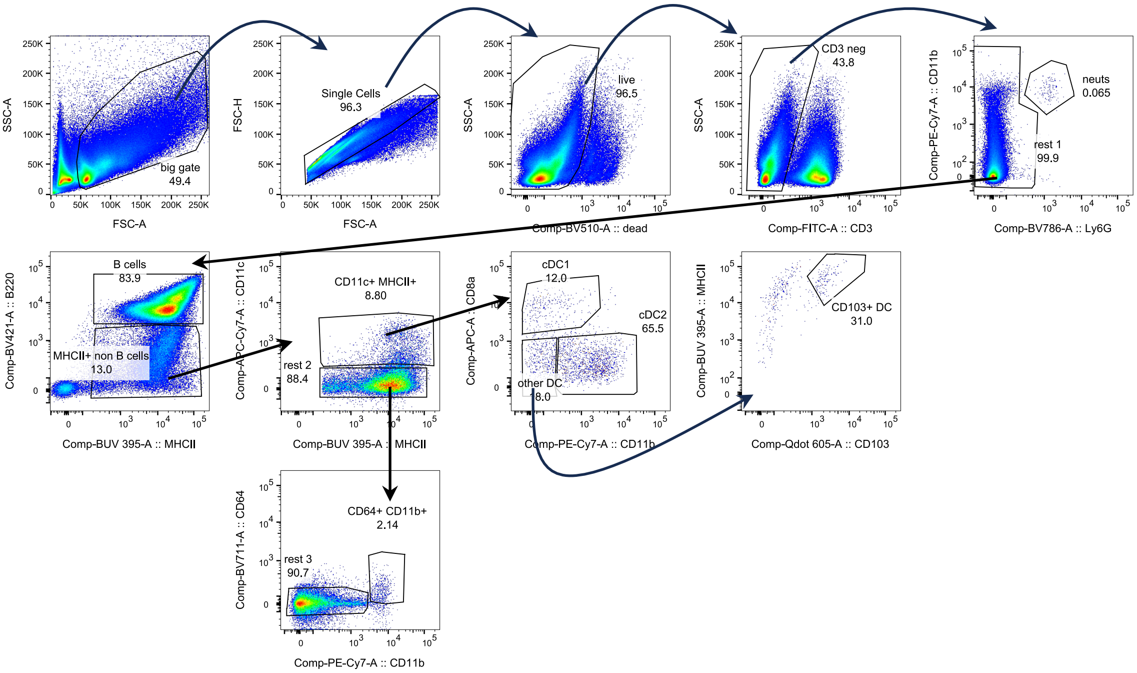

E

Spleen gating

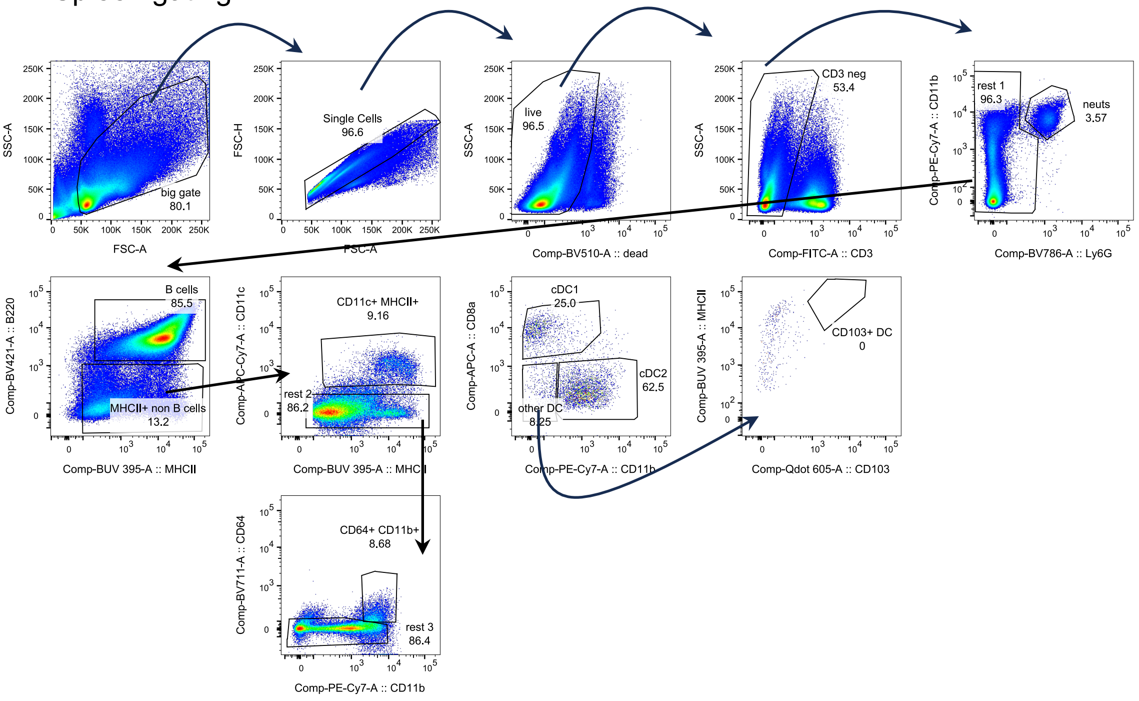

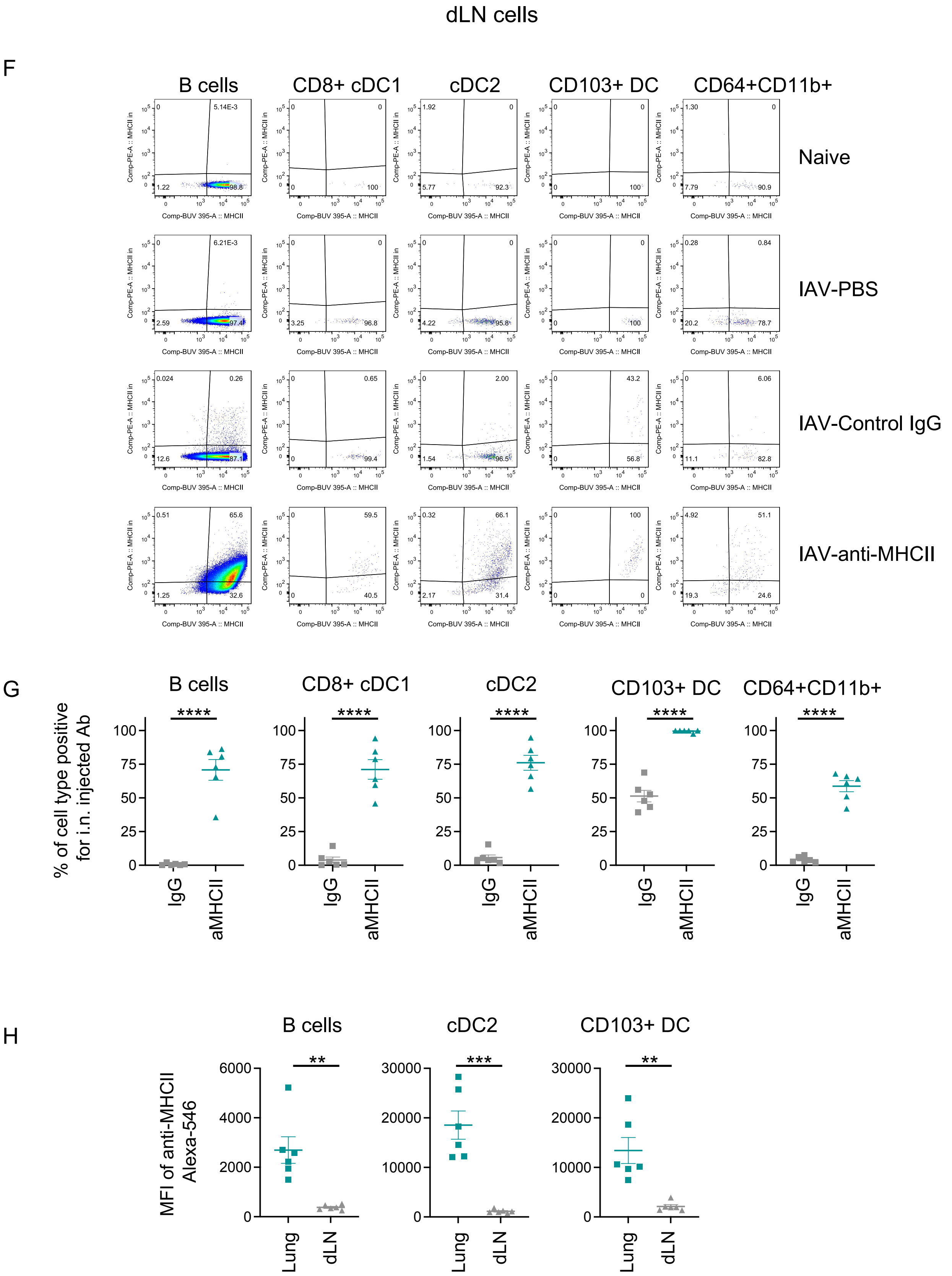
dLN cells
F
B cells
CD8+ cDC1
cDC2
CD103+ DC
CD64+CD11b+
Naive
IAV-PBS
IAV-Control IgG
IAV-anti-MHCII
Comp-PE-A :: MHCII in
Comp-BUV 395-A :: MHCII
G
% of cell type positive for i.n. injected Ab
IgG
aMHCII
H
MFI of anti-MHCII Alexa-546
Lung
dLN

Spleen cells

I

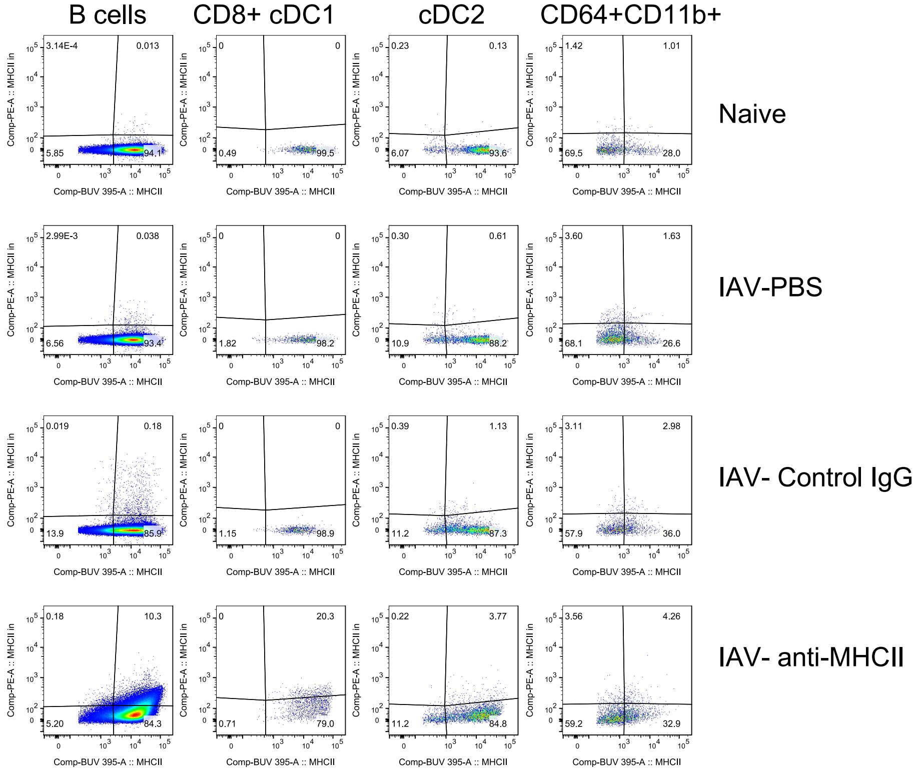

K

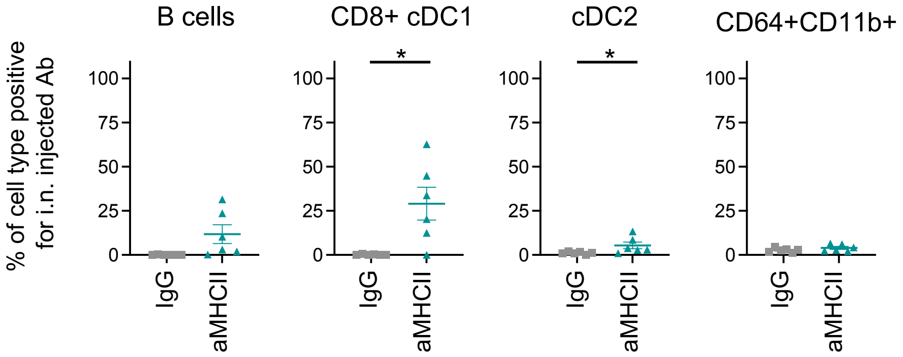

Supplementary Figure 6. Intranasal anti-MHCII binds to MHCII+ cells in the lung more robustly than to MHCII+ cells in the dLN or spleen 2 days post-instillation

C57BL/6 mice were infected with IAV i.n. on day 0 and received 100µg of either control IgG or anti-MHCII labelled with Alexa-Fluor546 i.n on day 6. 2 days later, single cell suspensions of lung (A-C), mediastinal lymph node cells (D, F-H), and spleen (E,I-K) were stained for flow cytometry. The gating strategy in SF5A was used to identify lung monocytes, macrophages, B cells, cDC1 and cDC2 and the strategies in SF6 D-E used to identify these cells in the dLN and spleen respectively. The data are representative of two experiments with a total of 6 (control IgG, 3/experiment) and 6 (anti-MHCII, 2 or 4/experiment) mice. In A, F and I the numbers show the percentages of cells within the quadrants.

In B, G and K, the percentages of the cell populations double positive for the i.n. antibody and anti-MHCII labelled *ex vivo* are shown. In C, the number of the indicated cell types in the lung are shown and H shows the MFI of the instilled anti-MHCII on the indicated populations. In these graphs, error bars are SEM and the horizontal line shows the mean of the group apart from in C where the number of macrophages and cDC2 are not normally distributed and the median with the interquartile range is shown, normality tested by Shapiro-Wilk tests, significance tested either by T-test or a Mann Whitney U test for the data that are not normally distributed, *:p<0.05, **:p<0.01 ***: p<0.001, ****:p<0.0001.

Supplementary Figure 7: Gating to identify MHC tetramer+ and EYFP+ T cells IAV specific CD4 T cells

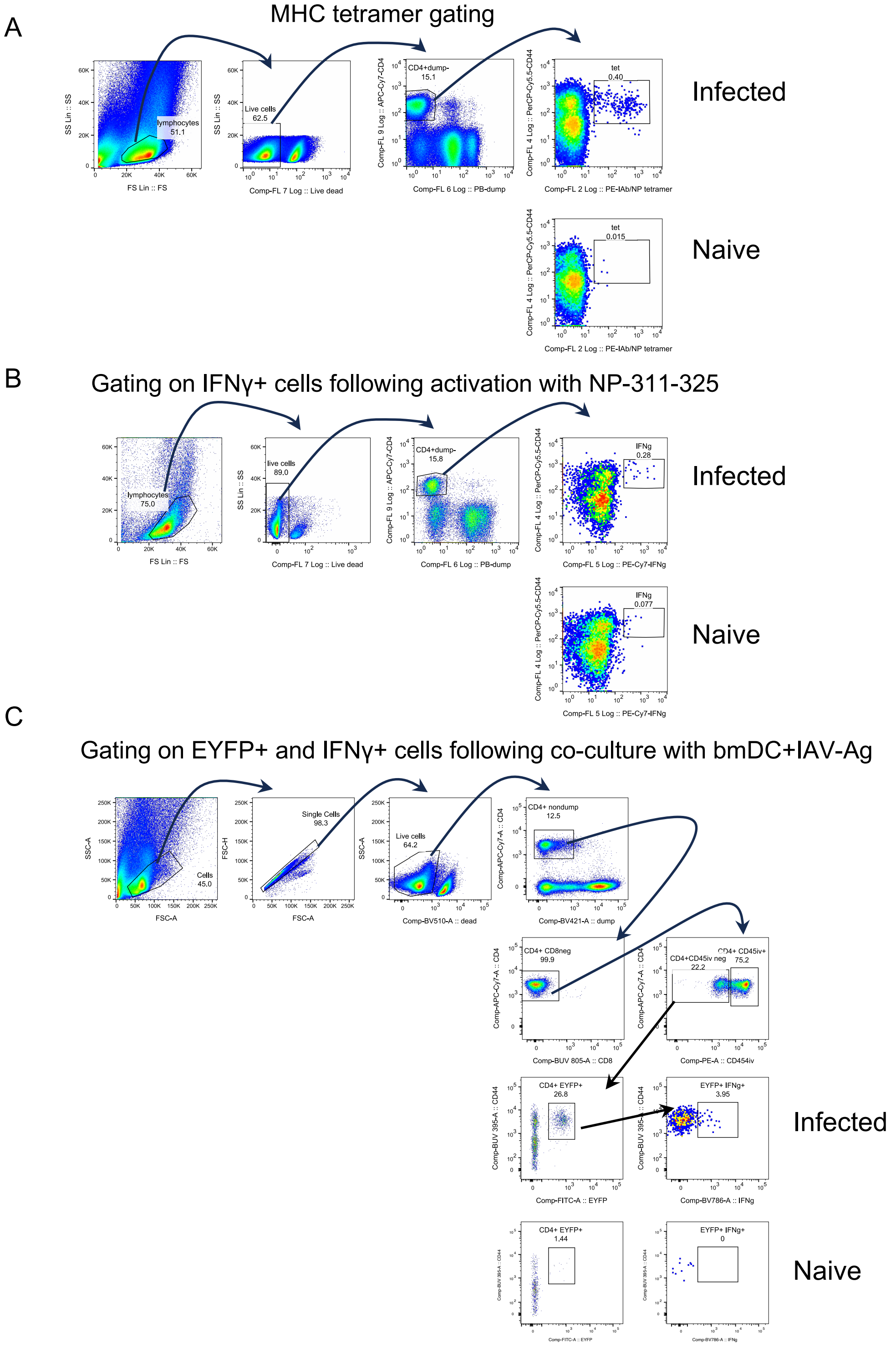

Supplementary Figure 7. Gating to identify MHC tetramer+ and EYFP+ T cells IAV specific CD4 T cells

C57BL/6 mice (A, B) or reporter TRACE mice (C) were infected with IAV i.n. on day 0 and received 100µg of either control IgG or anti-MHCII i.n on day 6 and 12. On day 40, single cell suspensions of lung cells were stained for flow cytometry either directly *ex vivo* (A), following 6 hours of *ex vivo* restimulation with either $NP_{311\text{-}325}$ peptide (B), or bone marrow derived DCs (bmDCs) incubated with IAV-Ag (C). All *ex vivo* restimulations were done in the presence of Golgi Plug. In A-B, mice were perfused with PBS-EDTA to remove cells within the blood; in C, blood cells were labelled with PE-labelled CD45 injected i.v. 3 minutes prior to euthanasia.

Supplementary Figure 8. Upstream gating for PD1 and ICOS staining

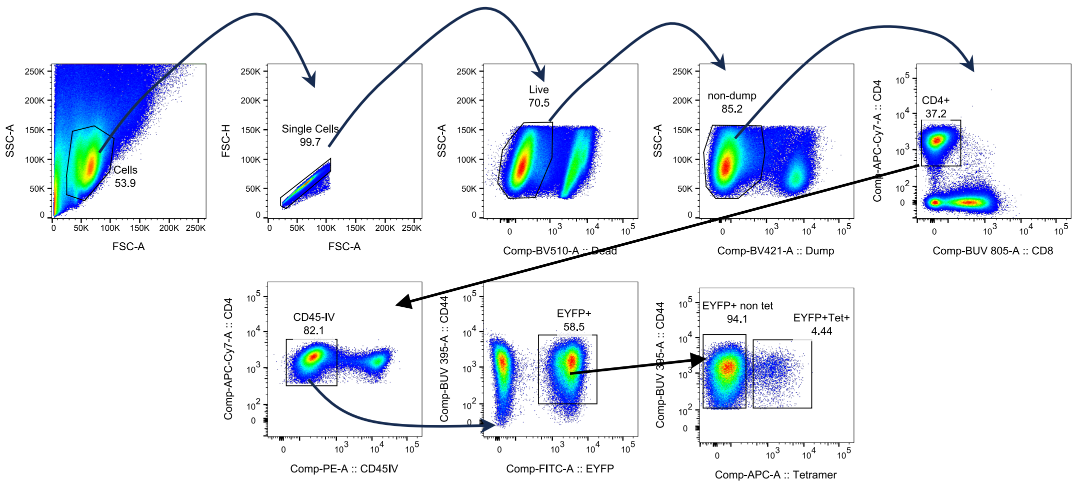

Supplementary Figure 8. Example upstream gating for PD1 and ICOS on IAV specific CD4 T cells

TRACE mice were infected i.n. with IAV on day 0 and 10 days later injected with CD45 PE i.v. 3minutes prior to removal of tissues. Single cell suspensions of the lung were stained with MHCII tetramer and surface antibodies and cells examined by flow cytometry. Cells are gated as shown and the numbers indicate the percentages of cells within the gates. Data are from the experiment shown in Figure 5.

Supplementary Figure 9. Memory MHC tetramer+ CD4 IAV specific T cells in the spleen and lymph node express higher levels of PD1 and ICOS than EYFP+ cells at day 10 but not day 40 post-infection

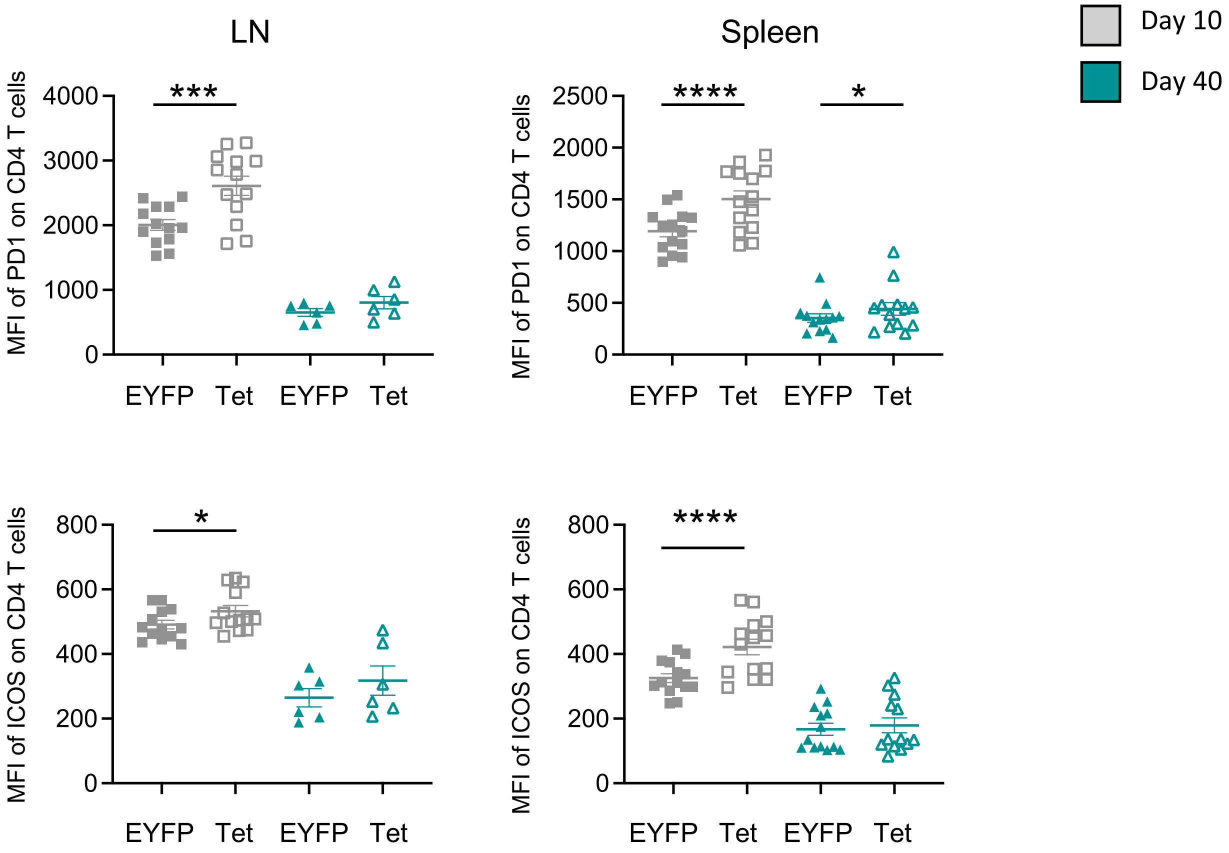

Supplementary Figure 9. MHC tetramer+ CD4 IAV specific T cells in the spleen and lymph node express higher levels of PD1 and ICOS than EYFP+ cells at day 10 but not day 40 post-infection

TRACE mice were infected i.n. with IAV on day 0, 10 or 40 days later these mice were injected with CD45 PE i.v. 3minutes prior to removal of tissues. Single cell suspensions of the spleen and draining lymph node were stained with MHCII tetramer, surface antibodies and viability dye and cells examined by flow cytometry. Cells are gated as shown in Supplementary Figure 7. Data are from two experiments with 5-8 mice per group: day 10: 4 or 7 mice/group; day 40: 4 or 8 mice/group. 2 lymph node samples were excluded from the first memory experiment and 5 from the second as less than 10 MHCII tetramer+ cells were present in the FACS plots. Error bars are SEM and differences between EYFP+ and MHCII tet+ cells tested using paired T-tests. *:p<0.05; ***:p<0.001; ****:p<0.0001.

Supplementary Figure 10: Anti-MHCII does not affect the expression of CD103, CD69 nor CXCR3 on EYFP+ CD4 T cells.

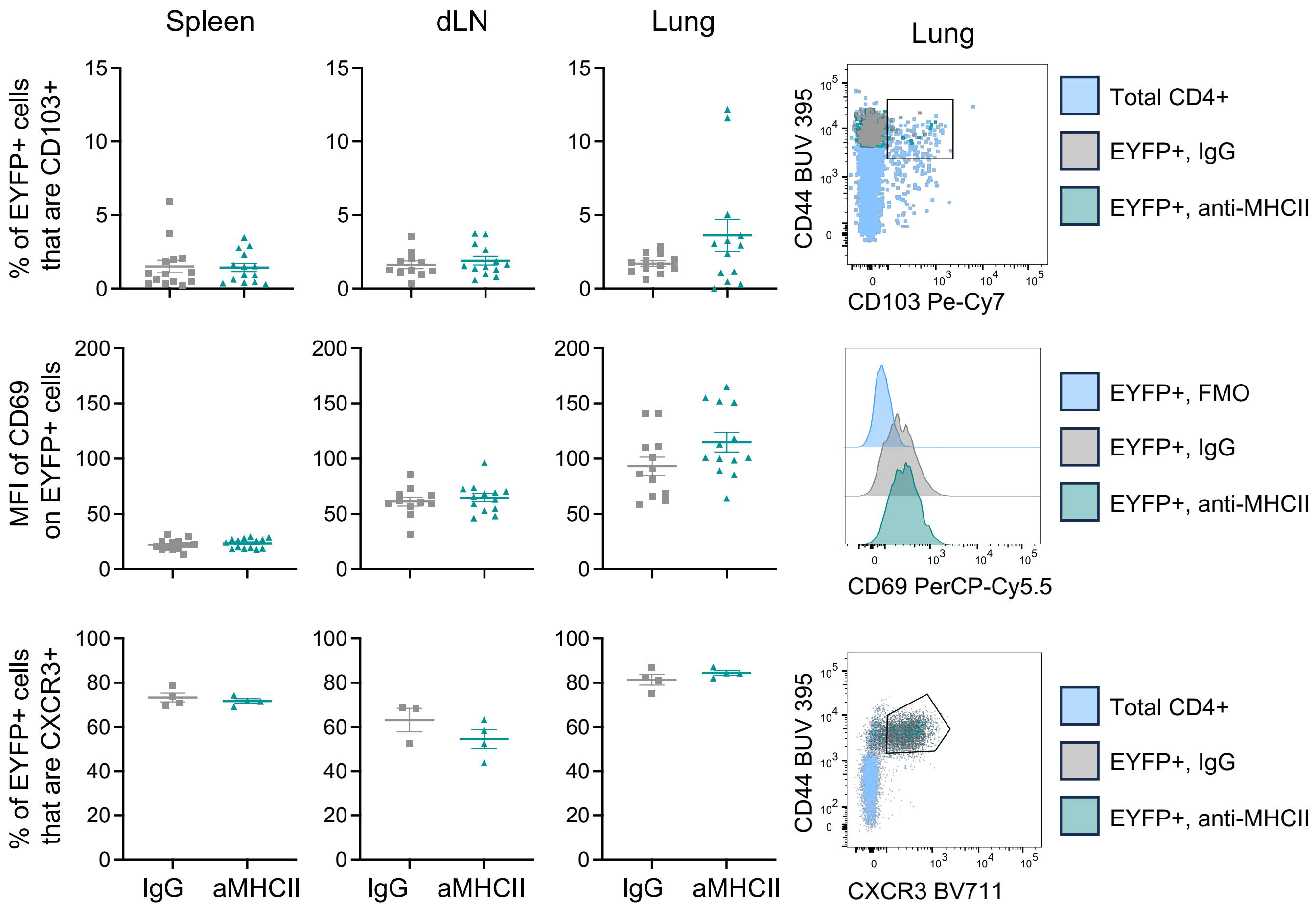

Supplementary Figure 10. Anti-MHCII does not affect the expression of CD103, CD69 nor CXCR3 on EYFP+ CD4 T cells.

TRACE mice were infected with IAV i.n. on day 0 and received 100µg of either control IgG or anti-MHCII i.n on day 6 and 12. On day 40, single cell suspensions of lymphoid or lung cells were stained for flow cytometry directly *ex vivo.* Cells are gated as in Supplementary Figure 7C on single, live lymphocytes that were CD45i.v. negative, CD4+ EYFP+ and negative for the MHCII and B220 or on CD4+ cells in the example flow plots for CD103 and CXCR3 staining, flow plots show lung CD4+ cells. The CD103 and CD69 data are from 3 experiments with 5 mice per group in two experiment and 4 mice per group in one experiment. For technical reasons, 4 lymph node and 4 lung samples are excluded from these data. The CXCR3 data are from one experiment with 4 or 5 mice per experiment. One lymph node sample is excluded for technical reasons.
